# A Mycovirus VIGS Vector Confers Hypovirulence to a Plant Pathogenic Fungus to Control Wheat FHB

**DOI:** 10.1002/advs.202302606

**Published:** 2023-08-16

**Authors:** Lihang Zhang, Shuangchao Wang, Shaojian Ruan, Clement Nzabanita, Yanfei Wang, Lihua Guo

**Affiliations:** ^1^ State Key Laboratory for Biology of Plant Diseases and Insect Pests Institute of Plant Protection Chinese Academy of Agricultural Sciences Beijing 100193 China

**Keywords:** FgGMTV1, hypovirulence, mycovirus, VIGS, wheat FHB

## Abstract

Mycovirus‐mediated hypovirulence has the potential to control fungal diseases. However, the availability of hypovirulence‐conferring mycoviruses for plant fungal disease control is limited as most fungal viruses are asymptomatic. In this study, the virus‐induced gene silencing (VIGS) vector p26‐D4 of *Fusarium graminearum* gemytripvirus 1 (FgGMTV1), a tripartite circular single‐stranded DNA mycovirus, is successfully constructed to convert the causal fungus of cereal Fusarium head blight (FHB) into a hypovirulent strain. p26‐D4, with an insert of a 75–150 bp fragment of the target reporter transgene transcript in both sense and antisense orientations, efficiently triggered gene silencing in *Fusarium graminearum*. Notably, the two hypovirulent strains, p26‐D4‐Tri101, and p26‐D4‐FgPP1, obtained by silencing the virulence‐related genes *Tri101* and *FgPP1* with p26‐D4, can be used as biocontrol agents to protect wheat from a fungal disease FHB and mycotoxin contamination at the field level. This study not only describes the first mycovirus‐derived VIGS system but also proves that the VIGS vector can be used to establish multiple hypovirulent strains to control pathogenic fungi.

## Graphical abstract

Fusarium head blight is a devastating fungal disease that affects wheat, barley, and oats worldwide. Here, the first mycovirus‐induced gene‐silencing vector is successfully constructed to convert pathogenic fungi into hypovirulent strains. The obtained hypovirulent strains can be used as biocontrol agents to control wheat FHB and mycotoxin contamination. This study provides a novel approach for the biocontrol of fungal diseases.

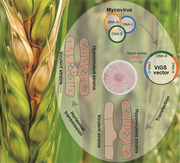

## 1 Introduction

Fungal viruses, known as mycoviruses, are ubiquitous in filamentous fungi, including plant, insect, and human pathogenic and beneficial fungi.^[^ ^1^ ^]^ Recently, mycoviruses have been rapidly identified from different fungal taxonomic groups owing to the development and low cost of high‐throughput nucleotide sequencing.^[^ ^2^ ^]^ The infection rates of mycoviruses in different fungal species range from nearly zero to greater than 90 percent.^[^ ^3^ ^]^ Mycovirus can be transmitted horizontally and vertically through hyphal anastomoses and spores, respectively.^[^ ^4^ ^]^ In addition, some mycoviruses such as Cryphonectria hypovirus 1 (CHV1) and Sclerotinia sclerotiorum hypovirulence‐associated DNA virus 1 (SsHADV‐1) can be hosted and transmitted by plants and insects, respectively.^[^ ^5^ ^]^ Mycoviruses have various genome types, including single‐stranded RNA genomes with positive and negative sense, double‐stranded RNA (dsRNA) genomes, and single‐stranded DNA (ssDNA) genomes.^[^ ^6^ ^]^ Most reported mycoviruses have RNA genomes, with a few known DNA genomes. Mixed infections by two or more mycoviruses in a single fungal strain are common and occasionally have a symbiotic relationship that benefits viral accumulation. The most typical representative sample showed the coexistence of yadokari virus 1 (YkV1) and an unrelated yadonushi virus 1 (YnV1). YkV1 cannot survive in the host alone and depends on YnV1 for replication and encapsidation in *Rosellinia necatrix*.^[^ ^7^ ^]^

Viruses severely threaten human health and cause significant economic losses in agriculture. However, viruses can also be used for biological control of harmful cellular organisms.^[^ ^1^, ^4^ ^]^ In agriculture, many pathogens and pests, such as *C. parasitica* and lepidopterans, have been controlled by different viruses.^[^ ^8^ ^]^ Although most viruses of economically significant plant pathogenic fungi do not affect host growth, spread, or pathogenicity, hypovirulence (attenuation of fungal virulence) causing mycoviruses have attracted much attention as potential candidates for the biocontrol of fungal diseases. Hypovirulences induced by two mycoviruses, CHV1 and SsHADV‐1, have been successfully used to biocontrol plant fungal diseases in the field.^[^ ^9^ ^]^ The hypovirulent *C. parasitica* strain containing CHV1 was developed as a biological control agent (BCA) for chestnut blight.^[^ ^5a^ ^]^ However, the use of natural hypovirulence delivered by CHV1 has been ineffective in controlling chestnut blight in the United States, possibly due to the high susceptibility of American chestnut to *C. parasitica* and the diversity of vegetatively incompatible groups of *C. parasitica* that restricts CHV1 spread.^[^ ^10^ ^]^ SsHADV‐1, the first mycovirus with a circular ssDNA genome, was successfully developed into a BCA.^[^ ^9^, ^11^ ^]^ SsHADV‐1 can not only protect plants from *S. sclerotiorum* infection but can also change the lifestyle of the fungal host from pathogenic to endophytic, thus stimulating plant growth and resisting infection by other pathogens.^[^ ^9^, ^12^ ^]^ In both cases, the biocontrol strategy exploited the hypovirulence caused by natural mycoviruses. Hundreds of fungal viruses have been identified, however, most mycoviruses remain latent and do not affect their hosts.^[^ ^13^ ^]^ For example, only four (Fusarium graminearum virus 1 strain DK21, Fusarium graminearum virus‐ch9, Fusarium graminearum hypovirus 2, and Fusarium oxysporum f. sp. dianthi mycovirus 1) among more than thirty mycoviruses in *Fusarium* spp., have adverse effects on fungal growth and pathogenicity.^[^ ^14^ ^]^ Therefore, the limited availability of mycoviruses with hypovirulent activity is a major challenge in mycovirus applications.

Virus‐induced gene silencing (VIGS) is an alternative technique based on post‐transcriptional gene silencing (PTGS) for gene function studies in plants. It is time‐saving and sometimes the only option in organisms where genetic modification methods are not mature.^[^ ^15^ ^]^ PTGS is a sequence‐specific RNA degradation mechanism triggered by dsRNA.^[^ ^16^ ^]^ In the cases of VIGS, endogenous gene transcripts homologous to fragments inserted into viral vectors are degraded by PTGS when the virus infects plant tissues and spreads systematically in the tissues.^[^ ^17^ ^]^ In addition to RNA viruses, several ssDNA geminiviruses have been successfully transformed into VIGS vectors.^[^ ^18^ ^]^ However, no mycovirus‐based VIGS vectors have been developed. Mycovirus‐based VIGS would not only be a powerful tool for fungal gene function analysis but could also be used to generate hypovirulent strains to control plant pathogenic fungi. In this study, biotechnological approaches such as VIGS were used to develop hypovirulent strains based on modified mycoviruses as novel biocontrol strategies for plant pathogenic fungi.

In our previous study, Fusarium graminearum gemytripvirus 1 (FgGMTV1), a tripartite circular ssDNA mycovirus, was isolated from *F. graminearum*, which causes Fusarium head blight (FHB) in wheat. FgGMTV1 is classified as a member of a new species, *Gemytripvirus fugra1*, in the family *Genomoviridae*. Each of the three genomic segments encodes a single protein; DNA‐A encodes a replication initiation protein (Rep), DNA‐B encodes a genomovirus‐like capsid protein (CP), and DNA‐C encodes a protein p26 of unknown function. Based on the successful construction of infectious clones containing a dimeric tandem repeat of DNA‐A, a dimeric tandem repeat of DNA‐B, and a 1.6‐meric tandem repeat of DNA‐C, the relationship between the three components was clarified. DNA‐A and DNA‐B are mutually interdependent for their replication and are associated with growth reduction and hypovirulence in the absence of DNA‐C. Co‐infection with DNA‐A, DNA‐B, and DNA‐C leads to latent infection, and DNA‐C is essential for latency.^[^ ^19^ ^]^

FHB is a devastating fungal disease caused by *Fusarium* spp. in wheat, barley, and oats. Furthermore, *F. graminearum* is the primary causative pathogen of wheat FHB in China, the USA, Canada, and Europe.^[^ ^20^ ^]^ With changes in climate and farming systems, wheat FHB outbreaks have recently become increasingly severe in these countries.^[^ ^21^ ^]^ In addition to direct yield loss, *F. graminearum* produces mycotoxins, such as trichothecenes and zearalenone, in host plants, threatening human and animal health. Trichothecene deoxynivalenol (DON), an important virulence factor in *F. graminearum*, is a mycotoxin that affects food and feed safety. Therefore, mycotoxins, particularly DON, are the prime targets for restricting the symptoms of FHB and increasing food safety. The control of FHB mainly relies on chemical fungicides, which pose the risks of environmental pollution and fungicide resistance.^[^ ^22^ ^]^

In this study, we constructed an FgGMTV1‐based VIGS vector, p26‐D4, and aimed to create a new BCA for FHB. We demonstrated that when p26‐D4 was modified to contain a 75–150 bp fragment of the reporter transgene transcript in the sense or antisense orientation, it could efficiently trigger gene silencing in *F. graminearum*. Furthermore, the hypovirulent fungal strains generated by FgGMTV1‐based VIGS vectors could be used to control FHB under field conditions. To our knowledge, this is the first report of a mycovirus‐derived VIGS vector. This mycovirus‐based VIGS system has great potential for investigating fungal gene function. In addition, we propose a new approach for mycovirus‐based fungal disease biocontrol using hypovirulent strains.

## 2 Results

### 2.1 Construction of an FgGMTV1‐Based VIGS Vector

**Figure 1:**
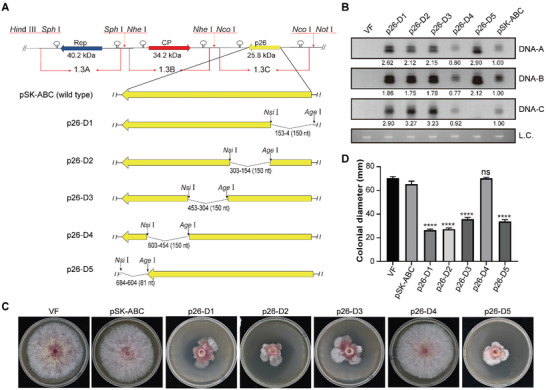
Schematic representation of DNA‐C deletion mutants and their infection activity. A) The genetic organization of the infectious clone, pSK‐ABC, is shown at the top. A partial repeat of DNA‐A (1.3‐mer tandem repeat), DNA‐B (1.3‐mer tandem repeat), and DNA‐C (1.5‐mer tandem repeat) were cloned into the vector pBluescript II SK (+) (pSK). 150 nt was deleted continually from the p26 encoding orientation. MCS, containing *Age* I and *Nsi* I sites, was introduced at two ends of deletion sites. Arrows indicate the orientation of ORFs. B) Viral genome accumulation in the deletion mutants. The DNA‐A, DNA‐B, and C components were blotted with probes A, B, and C, respectively. PH‐1 (virus‐free, VF) was used as the negative control, and the pSK‐ABC‐infected strain as the positive control. Fungal genomic DNA serves as the loading control (L.C.). The values of positive control samples were set as 1.00. This experiment was repeated thrice with similar results. Representative images are shown. C) Colony morphology of PH‐1 (VF) and strains infected by pSK‐ABC and p26‐D1‐D5 after 4 days of culture on PDA in the dark (*n* = 3). D) Comparison of the colonial diameter among PH‐1 (VF) and strains infected by pSK‐ABC and p26‐D1‐D5 (*n* = 3). Data are the mean of three independent biological replicates. Error bars represent standard deviation. An asterisk indicates a statistically significant difference according to the one‐way ANOVA (Dunnett's *post‐hoc* test). ^****^ *p* < 0.0001. ns indicates no significance.

Previously, we constructed infectious clones of FgGMTV1 using three vectors containing a dimer tandem repeat of DNA‐A, a dimer tandem repeat of DNA‐B, and a 1.6‐mer tandem repeat of DNA‐C.^[^ ^19a^ ^]^ In this study, to facilitate the molecular modification of FgGMTV1, we successfully cloned the three components of FgGMTV1 into a non‐integrated vector, pBluescript II SK (+) (pSK), named pSK‐ABC. It comprises a 1.3‐mer DNA‐A tandem repeat, a 1.3‐mer DNA‐B tandem repeat, and a 1.5‐mer DNA‐C tandem repeat, with multiple enzyme restriction sites between the three components (**Figure** **1**A). To test the infectivity of pSK‐ABC, transfectants were obtained by transfection of pSK‐ABC into *F. graminearum* PH‐1. Similar viral DNA accumulation was detected in the strain transfected with pSK‐ABC compared to the previous infectious clone of the three vectors (Figure S1A, Supporting Information). Furthermore, there were no significant differences in colony morphology, growth rate, or pathogenicity between fungal cultures transfected with plasmid vectors A+B+C and pSK‐ABC (Figure S1B–D, Supporting Information). These results confirmed that the optimized FgGMTV1 infectious clone, pSK‐ABC, has an infectious activity similar to that of the previous clone.

The VIGS vector is a well‐studied approach for fundamental research and agricultural applications.^[^ ^18a^ ^]^ Based on the successful construction and optimization of the infectious clone pSK‐ABC, FgGMTV1 has the potential to be modified into a VIGS vector. As DNA‐C is dispensable for FgGMTV1 replication and infection,^[^ ^19a^ ^]^ p26 coding region of DNA‐C was selected as a potential site for the insertion of a foreign gene. First, we constructed five consecutive p26 deletion mutants, p26‐D1 to D5, in which 150 nucleotides were sequentially deleted with the p26 coding orientation, and 81 nucleotides were deleted in mutant p26‐D5 at the 3′ end of the p26. Multiple cloning sites (MCS) were added to the two ends of the deletion sequence (Figure 1A). The infectivity and impact on host biology of the five viral deletion mutants were tested (Table S1, Supporting Information). A certain amount of viral DNA accumulation was detected in the five mutants by Southern blot analysis, indicating successful infection of the five mutants (Figure 1B). Sequencing analysis of the viral genome also confirmed the deletion of the corresponding DNA‐C sequence in each mutant, except for mutant p26‐D5. In addition, Southern blotting did not detect DNA‐C in the mutant p26‐D5, implying independent replication of the three components of FgGMTV1. However, there was a significant increase in DNA‐A, DNA‐B, and DNA‐C accumulation in the p26‐D1, p26‐D2, and p26‐D3 mutants. The accumulation of DNA‐A, DNA‐B, and DNA‐C in the strain infected with p26‐D4 was similar to that of the strain pSK‐ABC (Figure 1B). Additionally, mutants p26‐D1 to D3 and p26‐D5 exhibited >49.6% reduction in colony diameter and severe colony morphological abnormalities compared to strains PH‐1 or pSK‐ABC (Figure 1C,D). Moreover, as the culture was prolonged, the phenotypic changes of colony growth retardation and abnormal colony morphology were related to sporadic fungal sectorization with fluffy mycelial growth (Figure S2A, Supporting Information). Spontaneous viral clearance was observed in subcultures of these fast‐growing sectors (Figure S2B, Supporting Information), suggesting that the loss‐of‐function of DNA‐C tends to eliminate the virus from the infected cells. However, the p26‐D4 mutant showed stable viral presence in potato dextrose agar (PDA) fungal cultures with asymptomatic colony morphology of PH‐1 and pSK‐ABC (Figure 1C,D; Figure S2B, Supporting Information). Therefore, p26‐D4 was selected as a candidate for FgGMTV1‐based VIGS construction.

### 2.2 Functional Validation of the VIGS Vector Based on FgGMTV1 in *F. graminearum*

**Figure 2:**
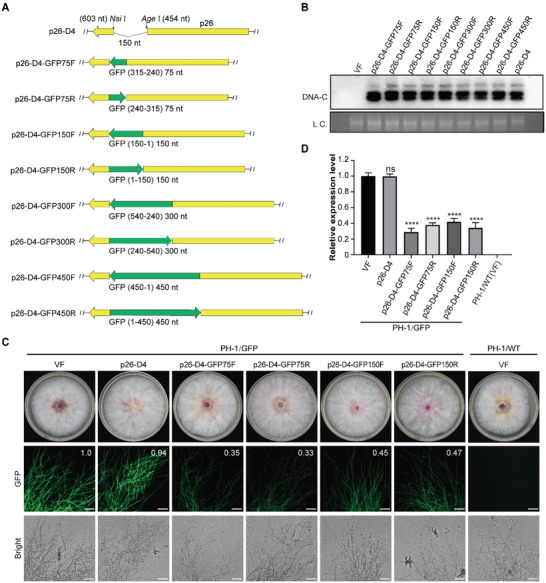
GFP silencing with the FgGMTV1‐based VIGS vector. A) Diagrams of the different cDNA fragments of the GFP gene in sense or antisense orientation were inserted into the MCS site of the p26‐D4 vector. Yellow and green arrows indicate the encoding orientation of p26 and GFP, respectively. F, sense orientation; R, antisense orientation. The numbers indicate the location of the GFP gene. B) Southern blot analysis of DNA extracted from mycelia of PH‐1/GFP (VF, as negative control), p26‐D4‐infected strain (as positive control), and the eight transfectants. Fungal genomic DNA serves as the loading control (L.C.). DNA‐C was detected with hybridization probe C. This experiment was repeated three times with similar results; representative images are shown. C) Colony morphology and green fluorescence intensity of strains infected by p26‐D4‐GFP75F/R, p26‐D4‐GFP150F/R, p26‐D4, and virus‐free strains of PH‐1/GFP and PH‐1/wild‐type(WT). Values in the respective panels were quantified using ImageJ, with the strain PH‐1/GFP (VF) expressed as 1.0. Scale bars, 100 µm. This experiment was repeated thrice with similar results. Representative images are shown. D) The relative expression level of the GFP gene in strains infected by p26‐D4‐GFP75F/R and p26‐D4‐GFP150F/R. qRT‐PCR was carried out with the *EF‐1α* transcript levels as an internal control (*n* = 3). Error bars represent standard deviation. An asterisk indicates a statistically significant difference according to the one‐way ANOVA (Dunnett's *post‐hoc* test). ^****^ *p* < 0.0001. ns indicates no significance.

Green fluorescent protein (GFP) is widely used as a reporter gene for VIGS in various plants. To explore whether the vector p26‐D4 could silence a gene in *F. graminearum*, we used the GFP transgenic *F. graminearum* PH‐1 strain (PH‐1/GFP) for GFP silencing. In this assay, we constructed eight VIGS vectors, p26‐D4‐GFP75F, p26‐D4‐GFP75R, p26‐D4‐GFP150F, p26‐D4‐GFP150R, p26‐D4‐GFP300F, p26‐D4‐GFP300R, p26‐D4‐GFP450F, and p26‐D4‐GFP450R, which carried different sizes of sense and antisense GFP, based on p26‐D4 (**Figure** **2**A; Table S1, Supporting Information). These vectors were transfected into the PH‐1/GFP strain via PEG‐mediated protoplast transformation. Southern blot analysis indicated that all vectors were infective (Figure 2B). Sequence analysis confirmed no mutations or deletions in the inserted fragments in transfectants infected with p26‐D4‐GFP75F/R and p26‐D4‐GFP150F/R. However, the GFP fragments inserted into the DNA‐C genomes of strains infected with p26‐D4‐GFP300F/R and p26‐D4‐GFP450F/R were partially or entirely lacking (Figure S3, Supporting Information). In addition, the four strains infected with p26‐D4‐GFP75F/R and p26‐D4‐GFP150F/R showed morphological characteristics similar to those of the PH‐1/GFP and p26‐D4‐infected strains (Figure 2C). These results suggest that the length of the inserted foreign gene fragment in p26‐D4 cannot exceed the length of the deleted viral sequence.

To verify the efficacy of gene silencing, we assessed the fluorescence intensity and transcriptional level of GFP in four strains infected with p26‐D4‐GFP75F/R and p26‐D4‐GFP150F/R. Compared to virus‐free and p26‐D4‐infected PH‐1/GFP strains, the intensity of green fluorescence of the VIGS strains was significantly reduced (p26‐D4‐GFP75F/R decreased by 65% and 67%, respectively, and p26‐D4‐GFP150F/R decreased by 55% and 53%, respectively) (Figure 2C). This result was further confirmed by RT‐qPCR analysis, which showed that homologous GFP mRNA levels in GFP‐silenced strains were reduced by 55–75% compared to virus‐free and p26‐D4‐infected strains of PH‐1/GFP (Figure 2D). In conclusion, 75 and 150 bp inserted FgGMTV1‐based VIGS vectors in both sense and antisense orientations, p26‐D4‐GFP75F/150F and p26‐D4‐GFP75R/150R, caused a GFP‐silencing phenotype in *F. graminearum*.

### 2.3 Hypovirulent Strains Obtained by the FgGMTV1‐based VIGS Vector

**Figure 3:**
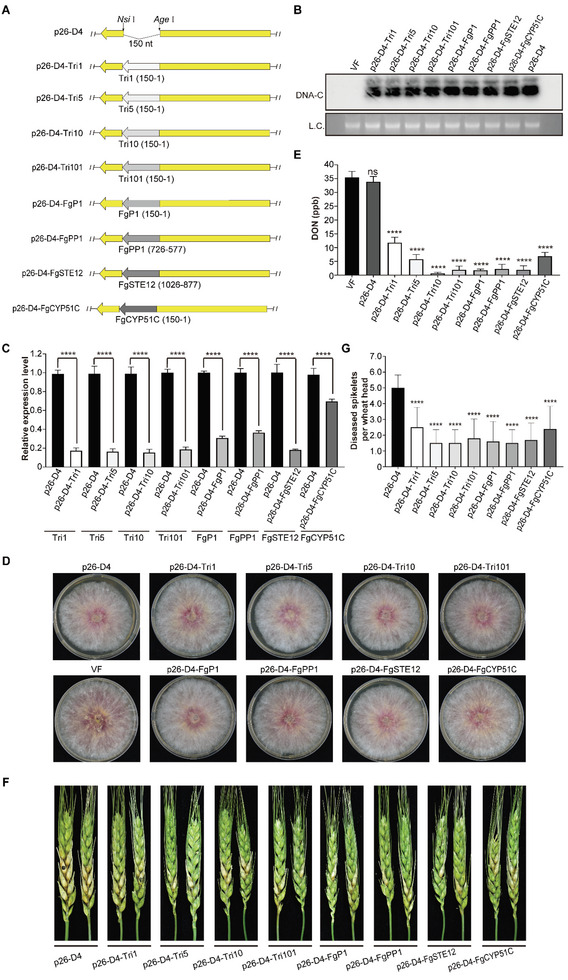
Silencing of endogenous genes with the FgGMTV1‐based VIGS vector in *F. graminearum*. A) Diagram of p26‐D4 inserted with 150 nt fragments of genes, *Tri1* (Accession: XM_011317365), *Tri5* (Accession: XM_011323870), *Tri10* (Accession: XM_011323869), *Tri101* (Accession: XM_011329426), *FgP1* (Accession: XM_011320233), *FgPP1* (Accession: XM_011328666), *FgSTE12* (Accession: XM_011328754), and *FgCYP51C* (Accession: XM_011327038). All genes were inserted in sense orientation into the MCS site of the vector p26‐D4. Yellow and grey arrows indicate the encoding orientation of p26 and target genes, respectively. The numbers indicate the position of inserted gene fragments. B) Viral genome accumulation in the transfectants. Southern blot analysis of DNAs extracted from mycelia of strain PH‐1 (VF, as negative control), eight transfectants, and p26‐D4‐infected strain (as positive control), respectively. The blots show the component of DNA‐C. Fungal genomic DNA serves as the loading control (L.C.). This experiment was repeated thrice with similar results. Representative images are shown. C) The expression level of target genes *Tri1*, *Tri5*, *Tri10*, *Tri101*, *FgP1*, *FgPP1*, *FgSTE12*, and *FgCYP51C* in strains infected with the corresponding VIGS vectors. These samples were sub‐cultured twice. qRT‐PCR was carried out with the *EF‐1α* transcript level as an internal control (*n* = 3). D) Colony morphology of the VIGS vector transfectants. The fungal strains were cultured on PDA plates for 4 days (*n* = 3). E) The production of DON in the VIGS vector transfectants. The DON production of each transfectant was determined after growth in mycotoxin induction medium (TBI) for three days (*n* = 3). F,G) FHB symptoms and the numbers of diseased spikelets per invaded wheat head caused by the VIGS vector transfectants. The number of diseased spikelets per invaded wheat head was counted at 12 dpi (*n* = 15). Error bars represent standard deviation. An asterisk indicates a statistically significant difference according to the one‐way ANOVA (Dunnett's *post‐hoc* test). ^****^ *p* < 0.0001.

Hypovirulent fungal strains are potential candidates for biological control of plant fungal diseases. The FgGMTV1 VIGS vector was designed to create multiple hypovirulent strains by silencing host pathogenicity‐related genes. Eight genes involved in trichothecene biosynthesis or pathogenicity of *F. graminearum* were selected for VIGS construction: *Tri1* (FGSG_00071), *Tri5* (FGSG_03537), *Tri10* (FGSG_03538), *Tri101* (FGSG_07896), *FgP1* (FGSG_12164), *FgPP1* (FGSG_07233), *FgSTE12* (FGSG_07310), and *FgCYP51C* (FGSG_11024) (Table S2, Supporting Information).^[^ ^23^ ^]^ VIGS vectors were constructed, each harboring 150 bp fragments of the corresponding eight genes in the sense orientation (**Figure** **3**A; Table S3, Supporting Information), and all transfectants were obtained by PEG‐mediated protoplast transfection (Table S1, Supporting Information). After two subcultures, Southern blot analysis revealed successful infection with the VIGS vector in eight transfectants (Figure 3B). qRT‐PCR analysis was performed on the eight transfectants to confirm the silencing efficiency of the eight target genes. The expression levels of the eight target genes in the transfectants were significantly inhibited compared with those in the strain infected with p26‐D4 (Figure 3C). In strains infected with p26‐D4‐Tri1, p26‐D4‐Tri5, p26‐D4‐Tri10, and p26‐D4‐Tri101, the expression levels of all related genes were reduced by more than 80%. In strains infected with p26‐D4‐FgP1, p26‐D4‐FgPP1, p26‐D4‐FgSTE12, and p26‐D4‐FgCYP51C, the expression of the associated genes decreased by at least 69%, 63%, 82%, and 32%, respectively (Figure 3C; Table S2, Supporting Information). To confirm the specificity of gene silencing, we measured the expression levels of eight target genes in the p26‐D4‐GFP150F‐infected strain using qRT‐PCR analysis. The results showed no significant differences in the expression levels of the eight target genes between the p26‐D4‐GFP150F‐infected strain and p26‐D4‐infected or virus‐free strains (Figure S4A, Supporting Information). Moreover, the genetic stability of foreign inserts in these recombinant viruses and the silencing efficacy of the target genes were evaluated by serial subcultures. The qPCR results showed that the target genes retained lower expression levels in the sixth subculture (Figure S5, Supporting Information). Collectively, the FgGMTV1‐based VIGS vector efficiently silenced the endogenous genes of *F. graminearum*.

As these eight genes were not associated with colony morphology and colony growth on PDA medium, strains carrying viral mutants with the gene fragments showed no significant phenotypic differences on PDA plates compared to the PH‐1 (VF) and p26‐D4‐infected strains (Figure 3D). To further examine whether the VIGS vectors inhibit the synthesis of mycotoxins, we evaluated the accumulation of DON in eight gene‐silenced transfectants. Compared with PH‐1 (VF) and p26‐D4‐infected strains, DON production in strains infected with p26‐D4‐Tri1, p26‐D4‐Tri5, p26‐D4‐Tri10, p26‐D4‐Tri101, p26‐D4‐FgP1, p26‐D4‐FgPP1, p26‐D4‐FgSTE12, and p26‐D4‐FgCYP51C was significantly decreased (Figure 3E), whereas DON production in the p26‐D4‐GFP150F‐infected strain did not change significantly (Figure S4B, Supporting Information). In addition, these eight genes significantly affect the virulence of *F. graminearum*. Therefore, we tested their virulence on flowering wheat heads. By applying small equisized mycelial plugs of eight transfectants, the number of diseased spikelets per wheat head decreased significantly 12 days post‐inoculation. The p26‐D4‐infected strain spread from the inoculated spikelet to other spikelets in the head, causing severe scab symptoms. However, the symptoms were limited to the inoculated or adjacent spikelets in strains infected with p26‐D4‐Tri1, p26‐D4‐Tri5, p26‐D4‐Tri10, p26‐D4‐Tri101, p26‐D4‐FgP1, p26‐D4‐FgPP1, p26‐D4‐FgSTE12, and p26‐D4‐FgCYP51C (Figure 3F,G). Altogether, these results indicate that the p26‐D4 VIGS vector could efficiently suppress the expression of targeted pathogenicity‐related genes in *F. graminearum*. In addition, the hypovirulent strains created using the VIGS vector can be used as potential biocontrol candidates.

### 2.4 VIGS‐Induced Hypovirulent Strains as Biocontrol Agents for FHB in Wheat

**Figure 4:**
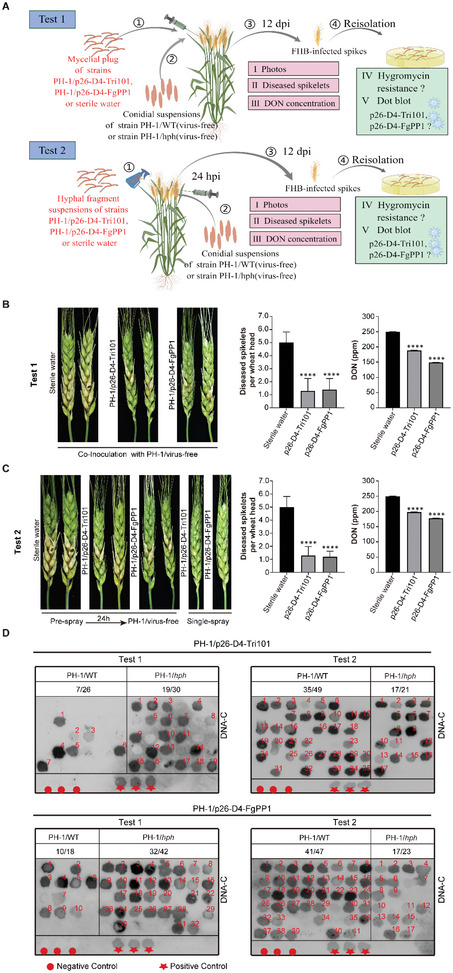
The control of FHB in wheat with hypovirulent strains containing the VIGS vector in the field. A) Experimental procedure to evaluate the biocontrol activity of the VIGS vector, p26‐D4‐Tri101 and p26‐D4‐FgPP1. Test 1: Small equisized mycelial plugs of the two hypovirulent strains were inoculated together with 10 µL of conidial suspension of strains PH‐1/WT(virus‐free) or PH‐1/*hph*(virus‐free) into the glume of a spikelet. Test 2: Hyphal fragment suspensions were sprayed on wheat spikes, followed by inoculation of 10 µL PH‐1/WT(virus‐free) or PH‐1/*hph*(virus‐free) conidial suspension at 24 h post spraying. Sterile water was used as the negative control. The illustration was made using Figdraw. B,C) FHB symptom, the number of diseased spikelets per invaded wheat head, and DON concentration in infected spikelets in Test 1 and Test 2 (*n* = 15). PH‐1/WT (virus‐free) was used as a virulent strain. The data was analyzed using GraphPad Prism version 8.0. Error bars represent standard deviation. An asterisk indicates a statistically significant difference according to the one‐way ANOVA (Dunnett's *post‐hoc* test). ^****^ *p* < 0.0001. D) Horizontal transmission analysis of recombinant viruses p26‐D4‐Tri101 and p26‐D4‐FgPP1 in Test 1 and Test 2. Fungal genomic DNA was extracted from PH‐1/WT and PH‐1/*hph* strains reisolated from the infected spikelets, and dot blot analysis was performed with Probe C. Red numbers indicate positive samples. Red circles and stars indicate negative (PH‐1, VF) and positive controls (pSK‐ABC‐infected strain), respectively.

In this study, we attempted to utilize hypovirulent strains generated using the VIGS vector to control FHB in wheat at the field level. Two hypovirulent strains, generated by p26‐D4‐Tri101 and p26‐D4‐FgPP1, were selected to test the biocontrol efficacy in wheat spikes by applying their small equisized mycelial plugs together with 10 µL of conidial suspension of virulent strain PH‐1 into the glume of a spikelet (**Figure** **4**A). After 12 days, the expansion of FHB was significantly decreased (*p* < 0.0001), with a reduced number of infected spikelets (Figure 4B). The protective levels in strains infected with p26‐D4‐Tri101 and p26‐D4‐FgPP1 were 74% and 72%, respectively (Figure 4B). DON is an important trichothecene mycotoxin produced by *F. graminearum*, and is positively correlated with the virulence of *F. graminearum*. We also analyzed the DON content of spikelets inoculated with hypovirulent strains. Compared to PH‐1 inoculation alone, DON accumulation was reduced by 25% or 40% by the combined inoculation of the virulent strain PH‐1 and the hypovirulent strains transfected with p26‐D4‐Tri101 or p26‐D4‐FgPP1, respectively (Figure 4B).

In addition, spraying p26‐D4‐Tri101‐ and p26‐D4‐FgPP1‐infected hyphal suspensions protected wheat against the virulent strain PH‐1 (Figure 4A). Briefly, suspensions of hyphal fragments of strains infected by p26‐D4‐Tri101 and p26‐D4‐FgPP1 were sprayed on wheat spikes, followed by inoculation of 10 µL PH‐1 conidial suspension 24 h post spraying. Sterile water was used as the negative control. Twelve days after inoculation, there was a significant difference (*p* < 0.0001) in the number of diseased spikelets per infected wheat head between each treatment group and the negative control group, whereas a single spraying of p26‐D4‐Tri101 or p26‐D4‐FgPP1‐infected hyphal fragment suspensions (≈1 × 10^5^ cfu mL^−1^) did not cause obvious scab symptoms in wheat spikes (Figure 4C). Pre‐spraying with hypovirulent strains infected with p26‐D4‐Tri101 (74% reduction, *p* < 0.0001) or p26‐D4‐FgPP1 (76% reduction, *p* < 0.0001) significantly inhibited the spread of PH‐1 to nearby spikelets (Figure 4C). Furthermore, DON production was significantly reduced in pre‐sprayed hypovirulent strains infected with p26‐D4‐Tri101 (21% reduction, *p* < 0.0001) or p26‐D4‐FgPP1 (29% reduction, *p* < 0.0001) (Figure 4C).

To verify whether the biocontrol ability of the hypovirulent strains was due to horizontal transmission of the mycovirus from the hypovirulent strain to the virulent strain through hyphal contact, we repeated Tests 1 and 2 under field conditions, using the virulent strain PH‐1 incorporating a hygromycin‐resistance gene (*hph*) (PH‐1/*hph*) instead of the wild‐type PH‐1 strain (PH‐1/WT) (Figure 4A), and obtained similar biocontrol effects as in the previous experiments (Figure S6A,B, Supporting Information). Twelve days after inoculation, we reisolated the fungi from the infected spikelets, evaluated them for hygromycin resistance, and performed dot blot analysis. The results showed that a high proportion of PH‐1/*hph* strains were infected with the recombinant viruses p26‐D4‐Tri101 and p26‐D4‐FgPP1 (63% for p26‐D4‐Tri101 and 76% for p26‐D4‐FgPP1 in Test 1, and 80% for p26‐D4‐Tri101 and 73% for p26‐D4‐FgPP1 in Test 2) (Figure 4D). In the corresponding wild‐type PH‐1 strains, the loss rates of the recombinant viruses were 73% for p26‐D4‐Tri101, 44% for p26‐D4‐FgPP1 in Test 1, and 29% for p26‐D4‐Tri101 and 13% for p26‐D4‐FgPP1 in Test 2 (Figure 4D). In total, 68% of the reisolated strains (140 PH‐1/WT and 116 PH‐1/*hph*) were infected with recombinant viruses (Figure 4D). The FgGMTV1‐based VIGS vector could likely be transmitted horizontally to virulent strains in the field. To further confirm the horizontal transmission of RNA silencing conferred by the p26‐D4‐based VIGS vector, fungal strains infected with p26‐D4‐GFP75F/R and virus‐free PH‐1/GFP (Geneticin‐resistant) were dual‐cultured on PDA plates (Figure S7A, Supporting Information). Southern blotting and sequencing analysis of subcultures of the recipient strain showed that the VIGS vector could spread horizontally from the donor (p26‐D4‐GFP75F/R‐infected strain) to the recipient (PH‐1/GFP) effectively (Figure S7B, Supporting Information). Compared with virus‐free PH‐1/GFP, the green fluorescence intensity in p26‐D4‐GFP75F/R‐infected recipient strains was reduced by 78–86% (Figure S7C, Supporting Information), and the expression level of GFP mRNA was 70% lower as determined by qPCR analysis (Figure S7D, Supporting Information). In conclusion, the VIGS vector can be horizontally transmitted from hypovirulent to pathogenic strains under laboratory and field conditions.

## 3 Discussion

In this study, we optimized the construction of the infectious clone of FgGMTV1, and developed a VIGS vector suitable for creating hypovirulent strains of *F. graminearum*. The availability of the FgGMTV1‐based VIGS vector enables silencing genes of interest to investigate their functions in *F. graminearum*. This is the first report of the successful construction and application of a VIGS vector based on a fungal virus. Notably, we showed that the FgGMTV1‐based VIGS vector could be manipulated to silence *Tri101* and *FgPP1*, virulence genes of *F. graminearum*, to control FHB in wheat. These characteristics enable the possibility of practical biocontrol using the FgGMTV1‐based VIGS vector.

Pathogenicity‐attenuating fungal viruses can be developed as potential biological control agents against pathogenic fungi in agriculture and medicine. However, the application of fungal viruses that cause host hypovirulence as BCA, is limited by their small number and narrow host range.^[^ ^1^ ^]^ The biotechnological approaches, such as VIGS and CRISPR/Cas, developed from viral infectious clones can extend the host range and accurately interfere with the pathogenicity and life cycle of the host.^[^ ^18b^ ^]^ Here, we developed an FgGMTV1‐based VIGS vector, p26‐D4, which connects tandem repeats of the three components of FgGMTV1 to an unintegrated vector, pSK (Figure 1A). Unit‐length geminiviral genomes are released from tandem genome repeats via rolling‐circle replication.^[^ ^24^ ^]^ To simplify genetic engineering, we used *Nsi* I and *Age* I restriction sequences to replace the 150 bp genome sequence of DNA‐C to insert target gene fragments. This strategy did not influence the functional expression of DNA‐C (Figure 1A). However, compared to the p26‐D4‐infected strain, mutants p26‐D1 to D3 and p26‐D5 exhibited reduced mycelial growth, severely abnormal colony morphology, significantly increased viral DNA accumulation (Figure 1B), and spontaneous viral clearance in the fungal culture on PDA (Figure S2B, Supporting Information), suggesting that they appeared to have lost DNA‐C or the function of DNA‐C to recover abnormal fungal phenotypes.^[^ ^19a^ ^]^ High levels of viral DNA appear to determine host growth impairment and spontaneous viral clearance. However, these deletion mutants can only be used as references to identify the key regions of DNA‐C responsible for symptom mitigation. Recent studies have shown that geminiviruses with Reps similar to genomoviruses encode additional small proteins with specific subcellular localization and potential virulence functions,^[^ ^25^ ^]^ and geminiviruses are known to encode viral proteins in bidirectional and partially overlapping ORFs.^[^ ^26^ ^]^ Therefore, point mutants specific to p26 or additional small proteins that may be encoded are needed to uncover the key sites for DNA‐C to recover abnormal fungal phenotypes.

Furthermore, we examined the RNA silencing efficiency and genetic stability of different fragment lengths inserted into the DNA‐C of FgGMTV1. An insertion shorter than 150 bp (the length of the deleted sequence) did not affect the genetic stability of the vector (Figure S3, Supporting Information). The small inserts for the FgGMTV1‐based VIGS vector p26‐D4 showed high efficiency in silencing endogenous genes such as *Tri1* (FGSG_00071), *Tri5* (FGSG_03537), *Tri10* (FGSG_03538), *Tri101* (FGSG_07896), *FgP1* (FGSG_12164), *FgPP1* (FGSG_07233), *FgSTE12* (FGSG_07310), and *FgCYP51C* (FGSG_11024) in *F. graminearum* (Figure 3C). In the case of ssDNA geminiviruses, bidirectional transcripts have been proposed as dsRNA precursors of primary viral short‐interfering RNAs (vsiRNAs), which are synthesized by the transcription of circular double‐stranded DNA minichromosomes. In *Arabidopsis thaliana*, DICER (DCL) 2 and DCL 4 recognize and cleave dsRNA segments into 22‐nt and 21‐nt vsiRNAs, respectively. The RNA‐induced silencing complex, containing ARGONAUTE (AGO) 1, cleaves viral and endogenous target gene mRNAs and inhibits their translation.^[^ ^17^, ^27^ ^]^ In our previous study, a large amount of 18–30 nt vsiRNAs were detected in a PH‐1 strain infected with FgGMTV1, suggesting that FgGMTV1 triggers host RNA silencing to defend against the virus.^[^ ^28^ ^]^ *F. graminearum* encodes two DCL and two AGO proteins,^[^ ^29^ ^]^ and their role in defense against the DNA virus FgGMTV1 requires further investigation. Additionally, the genetic stability of the inserted foreign gene fragment in these recombinant viruses and the silencing efficacy of the target gene were maintained for at least six subcultures (Figure S5, Supporting Information). Notably, the hypovirus CHV1‐EP713 has been successfully modified into expression vectors in the chestnut blight fungus *C. parasitica*,^[^ ^30^ ^]^ however, EGFP expression was stable only in silencing‐deficient mutants.^[^ ^31^ ^]^ In this study, the VIGS vector maintained its stability and persisted with high silencing efficacy, possibly due to differences in the RNA/DNA viral genome or host. However, the FgGMTV1‐based vectors could not tolerate inserts larger than the deleted lengths (Figure S3, Supporting Information). Long foreign inserts may influence the packing of FgGMTV1 virions. Other strategies could be attempted, such as duplicating DNA‐B or DNA‐C while completely replacing the CP or p26 proteins.

FHB, a fungal disease caused by *Fusarium* species that produce food toxins, currently devastates wheat production worldwide; however, few resistance resources have been discovered in the wheat germplasm.^[^ ^32^ ^]^ Eco‐friendly alternatives for crop protection will be the future trend. Here, we tested the possibility of biological control of FHB using a mycovirus‐based VIGS vector. We generated a variety of hypovirulent strains that can be used as potential biocontrol candidates. Notably, strains infected with p26‐D4‐Tri101 and p26‐D4‐FgPP1 successfully restricted the spread of the wild‐type virulent strain PH‐1, the causal agent of FHB in wheat, from the inoculation sites to nearby spikelets, using two different methods (Figure 4B,C). We also reisolated the fungi from the infected spikelets and performed dot blot analysis. Our results show that VIGS based on FgGMTV1 could be effectively transmitted to virulent strains through hyphal anastomosis in the field (Figure 4D).

In addition, hypovirulence induced by natural mycoviruses is associated with phenotypic changes, such as reduced pigmentation, sporulation, and growth defects.^[^ ^33^ ^]^ Therefore, large‐scale industrial production of hypovirulent strains is limited. Herein, the descriptive tools could choose host genes that reduce virulence but do not significantly influence vegetative growth. Moreover, the target sequences can be optimized to a shorter length, and one vector can simultaneously target multiple targets. Additionally, if transfected into a new host, the VIGS vector based on FgGMTV1 could be further applied to control different fungal diseases. This study demonstrated that the approach and vector for FHB biocontrol could be used to create multiple hypovirulent strains to control plant pathogenic fungi.

## 4 Experimental Section

### Fungal Strains and Culture Conditions

All fungal strains used in this study are listed in Table S1 (Supporting Information), and were cultured on PDA medium for 3–5 days at 25 °C for morphological observation or on cellophane‐covered PDA medium for DNA and RNA extractions. PH‐1 was used as the wild‐type strain of *F. graminearum*.^[^ ^34^ ^]^ PH‐1/GFP carrying the GFP and G418 resistance genes served as receptor strains for the VIGS target. Carboxymethyl cellulose liquid medium was used for conidiation assays. To reisolate *F. graminearum* from wheat grain, the diseased spikelet was sterilized with 70% alcohol, placed on PDA plates containing ampicillin, and incubated for 3 to 5 days at 25 °C. The regenerated colonies were transferred to Petri dishes containing PDA for subculture and molecular analysis.

### Plasmid Construction

The DNA sequences of the 1.3‐mer tandem repeat of DNA‐A, 1.3‐mer tandem repeat of DNA‐B, and 1.5‐mer tandem repeat of DNA‐C were produced by oligonucleotide synthesis (Tsingke Biological Technology, Beijing, China). To construct the infectious clones, restriction sites (*Hin*d III, *Sph* I, *Nhe* I, *Nco* I, and *Not* I) were incorporated between the three components, DNA‐A, DNA‐B, and DNA‐C. The products were inserted into the pSK vector and named the infectious clone pSK‐ABC (Figure 1A).

To construct the p26‐D1‐D5 deletion mutants (Figure 1A), 150 or 81 base pairs were sequentially deleted with the p26 coding orientation, and *Nsi* I and *Age* I sites were introduced using a KOD‐Plus‐Mutagenesis Kit (Toyobo, Osaka, Japan) based on the plasmid pSK‐ABC.

To test the silencing effect and tolerance of the p26‐D4 vector, the 75, 150, 300, or 450 nt sequences of the GFP gene were amplified from the plasmid pGTN and inserted into the p26‐D4 vector digested with *Nsi* I and *Age* I, and p26‐D4‐GFP75F (sense), p26‐D4‐GFP75R (antisense), p26‐D4‐GFP150F (sense), p26‐D4‐GFP150R (antisense), p26‐D4‐GFP300F (sense), p26‐D4‐GFP300R (antisense), p26‐D4‐GFP450F (sense), and p26‐D4‐GFP450R (antisense) plasmids were produced.

The gene fragments *Tri1* (sense), *Tri5* (sense), *Tri10* (sense), *Tri101* (sense), *FgP1* (sense), *FgPP1* (sense), *FgCYP51C* (sense), and *FgSTE12* (sense) were amplified from the total RNA of PH‐1 using RT‐PCR (Table S3, Supporting Information). The viral vectors p26‐D4‐Tri1, p26‐D4‐Tri5, p26‐D4‐Tri10, p26‐D4‐Tri101, p26‐D4‐FgP1, p26‐D4‐FgPP1, p26‐D4‐FgSTE12, and p26‐D4‐FgCYP51C were constructed using a procedure similar to that described above. The primers used in this study are listed in Table S4 (Supporting Information), and all constructs were confirmed by sequencing.

### Protoplast Preparation and Transfection Assays

Protoplast preparation of *F. graminearum* strains and transfection of virus‐infectious clones were performed following a previously described method with minor modifications.^[^ ^19a^ ^]^ Briefly, germinated mycelia were obtained by culturing fresh conidia (≈1×10^7^ conidia mL^−1^) overnight in Yeast Extract Peptone Dextrose liquid medium (yeast extract (5 g L^−1^), microbiological peptone (3 g L^−1^), and dextrose (10 g L^−1^)). The culture was harvested with three layers of lens paper and washed with sterile distilled water followed by 1.2 m KCl buffer. The cell walls of fresh mycelia were digested using 1.2 m KCl buffer containing 2.5% (w/v) driselase, 1% (w/v) lyticase, and 1% (w/v) snailase (Solarbio Science & Technology, Beijing, China). Protoplasts were filtered through three layers of lens paper and washed with STC buffer (1 M sorbitol, 50 mm Tris‐HCl pH 8.0, and 50 mm CaCl_2_∙2H_2_O).

For transfection, 5 µg (≈20 µL) of infectious clone plasmids were mixed with 200 µL protoplasts and incubated on ice for 30 min. Then, 1.5 mL PTC buffer (STC buffer containing 40% polyethylene glycol 4000) was added dropwise to the mixture of protoplast and plasmids, and incubated on ice for 30 min. Following incubation, 500 µL protoplast suspension was transferred to 9‐cm Petri dishes, embedded in 15 mL of warm TB3 solid medium, and cultured for 1–2 days at 25 °C in the dark. Fresh mycelial plugs were randomly selected and transferred onto PDA plates.

### DNA and RNA Extraction, PCR, Southern Blot, and RT‐qPCR

Genomic DNA was extracted from fungi using the cetyltrimethylammonium bromide method. A partial Rep fragment of 359 bp (probe A), a partial CP fragment of 335 bp (probe B), and a complete p26 coding region of 687 bp (probe C) were labeled with digoxigenin (DIG) by PCR amplification to specifically detect the accumulation of DNA‐A, DNA‐B, and DNA‐C. Southern blotting was used to detect viral infections. Probe preparation, hybridization, and signal detection were performed using DIG‐High Prime DNA Labeling and Detection Starter Kit II (Roche, Basel, Switzerland). RNA was extracted using FastPure Universal Plant Total RNA Isolation Kit (Nanjing Vazyme Biotech, Nanjing, China), and cDNA synthesis was carried out using Hifair III 1st Strand cDNA Synthesis Kit (Yeasen Biotechnology (Shanghai) Co., Ltd., Shanghai, China). Real‐time qPCRs were performed using a QuantStudio 5 (ABI) with Hieff qPCR SYBR Green Master Mix (Yeasen Biotechnology (Shanghai) Co., Ltd., Shanghai, China). *FgEF‐1α* was used as an internal control. Primer pairs used in this study are listed in Table S4 (Supporting Information).

### Observation of GFP Fluorescence

Sterile glass coverslips (22 × 22 mm) were placed on cellophane overlaying PDA, and a small mycelial plug was inserted at the edge of the coverslip. The hyphae of the transfected fungal colonies were allowed to grow onto the coverslips. Coverslips with attached hyphae were transferred to a glass slide, and GFP fluorescence was observed using a microscope (Carl Zeiss).^[^ ^30^ ^]^ The intensity of GFP green fluorescence was quantified using ImageJ software.

### Viral Horizontal Transmission via Hyphal Anastomosis

To assess the possibility of the horizontal transmission of the FgGMTV1‐based VIGS vector among strains of *F. graminearum*, fresh mycelial agar discs from p26‐D4‐GFP75F/R‐infected wild‐type PH‐1 strains and virus‐free PH‐1/GFP strains were dual‐cultured on PDA plates (9 cm in diameter). The p26‐D4‐GFP75F/R‐infected wild‐type PH‐1 strains served as donors, whereas virus‐free PH‐1/GFP strains served as recipients. After 4 days of dual culture, mycelial derivative isolates were obtained from the growth side of the recipients and transferred into fresh PDA plates containing G418 (200 µg ml^−1^). New colonies were subcultured on G418‐containing PDA plates and transferred onto fresh PDA plates for viral detection and fluorescence imaging.

### Pathogenicity, Toxisome Induction, and DON Production Assays

To assess the pathogenicity in flowering wheat heads, a field experiment was conducted using a susceptible wheat cultivar (Yangmai158). The biocontrol efficacy experiments are shown in Figure 4A. Briefly, in Test 1, a small equisized mycelial plug of strains infected by p26‐D4‐Tri101 or p26‐D4‐FgPP1 and 10 µL of conidial suspension (≈3 × 10^5^ conidia mL^−1^) of strain PH‐1 were inoculated together into the glume of a spikelet. In Test 2, hyphal fragment suspensions of p26‐D4‐Tri101 and p26‐D4‐FgPP1 infected strains (≈1 × 10^5^ cfu mL^−1^) were sprayed on wheat spikes, followed by inoculation of 10 µL PH‐1 conidial suspension at 24 h post spraying. Sterile water was used as the negative control. Fifteen replicates were performed for each strain. Twelve days post‐inoculation, the infected spikelets in each inoculated wheat head were recorded and collected. The DON concentration was analyzed using ultra‐performance liquid chromatography. To quantify DON production in the hypovirulent strains, each strain was cultured in liquid TBI at 28°C for three days in the dark, and DON Quantification Kit HEM1896 (HUANAN MAGNECH, Beijing, China) was used to quantify the DON production.

### Statistical Analysis

Data are represented as the mean ± standard deviation (SD). Statistical analysis were performed using GraphPad Prism version 8.0. Significant differences between the control and treatment groups were analyzed using the one‐way analysis of variance (ANOVA) followed by Dunnett's *post‐hoc* test for pairwise comparisons. Statistical significance was set at *p* < 0.05 (^*^ *p* < 0.05, ^**^ *p* < 0.01, ^***^ *p* < 0.001, and ^****^ *p* < 0.0001). The sample size (n) for each statistical analysis were reported in the corresponding “figure legends.”

## Conflict of Interest

The authors declare no conflict of interest.

## Supporting information

Supporting Information

## Data Availability

The data that support the findings of this study are available in the supplementary material of this article.

## References

[1] J. Wagemans , D. Holtappels , E. Vainio , M. Rabiey , C. Marzachì , S. Herrero , M. Ravanbakhsh , C. C. Tebbe , M. Ogliastro , M. A. Ayllón , M. Turina , Annu. Rev. Phytopathol. 2022, 60, 21. 35300520 10.1146/annurev-phyto-021621-114208

[2] H. Kondo , L. Botella , N. Suzuki , Annu. Rev. Phytopathol. 2022, 60, 307. 35609970 10.1146/annurev-phyto-021621-122122

[3] a) B. I. Hillman , A. Annisa , N. Suzuki , Adv. Virus. Res. 2018, 100, 99; 29551145 10.1016/bs.aivir.2017.10.003

[4] a) M. N. Pearson , R. E. Beever , B. Boine , K. Arthur , Mol. Plant. Pathol. 2009, 10, 115; 19161358 10.1111/j.1364-3703.2008.00503.x PMC6640375

[5] a) D. Rigling , S. Prospero , Mol. Plant Pathol. 2018, 19, 7; 28142223 10.1111/mpp.12542 PMC6638123

[6] a) J. M. Myers , T. Y. James , Curr. Biol. 2022, 32, R150; 35231405 10.1016/j.cub.2022.01.049

[7] R. Zhang , S. Hisano , A. Tani , H. Kondo , S. Kanematsu , N. Suzuki , Nat. Microbiol. 2016, 1, 15001. 27571749 10.1038/nmicrobiol.2015.1

[8] a) S. L. Anagnostakis , Science 1982, 215, 466; 17771259 10.1126/science.215.4532.466

[9] a) P. Zamora , A. B. Martín , R. San Martín , P. Martínez‐Álvarez , J. J. Diez , Biol. Control 2014, 79, 58;

[10] M. G. Milgroom , P. Cortesi , Annu. Rev. Phytopathol. 2004, 42, 311. 15283669 10.1146/annurev.phyto.42.040803.140325

[11] X. Yu , B. Li , Y. Fu , D. Jiang , S. A. Ghabrialc , G. Li , Y. Peng , J. Xie , J. Cheng , J. Huang , X. Yi , Proc. Natl. Acad. Sci. USA 2010, 107, 8387. 20404139 10.1073/pnas.0913535107 PMC2889581

[12] a) H. Zhang , J. Xie , Y. Fu , J. Cheng , Z. Qu , Z. Zhao , S. Cheng , T. Chen , B. Li , Q. Wang , X. Liu , B. Tian , D. B. Collinge , D. Jiang , Mol. Plant. 2020, 13, 1420; 32998002 10.1016/j.molp.2020.08.016

[13] I. T. Bocos‐Asenjo , J. Nino‐Sanchez , M. Ginesy , J. J. Diez , Int. J. Mol. Sci. 2022, 23, 9236. 36012499 10.3390/ijms23169236 PMC9409477

[14] a) P. Li , P. Bhattacharjee , S. Wang , L. Zhang , I. Ahmed , L. Guo , Front. Cell Infect. Microbiol. 2019, 9, 257; 31380300 10.3389/fcimb.2019.00257 PMC6657619

[15] D. Robertson , Annu. Rev. Plant Biol. 2004, 55, 495. 15377229 10.1146/annurev.arplant.55.031903.141803

[16] a) V. Ramachandran , X. Chen , Trends Plant Sci. 2008, 13, 368; 18501663 10.1016/j.tplants.2008.03.008 PMC2569976

[17] C. Rossner , D. Lotz , A. Becker , Annu. Rev. Plant Biol. 2022, 73, 703. 35138878 10.1146/annurev-arplant-102820-020542

[18] a) C. Huang , Y. Qian , Z. Li , X. Zhou , Sci. China Life Sci. 2012, 55, 99; 22415680 10.1007/s11427-012-4280-4

[19] a) P. Li , S. Wang , L. Zhang , D. Qiu , X. Zhou , L. Guo , Sci. Adv. 2020, 6, eaay9634; 32284975 10.1126/sciadv.aay9634 PMC7138691

[20] A. Chen , T. Islam , Z. Ma , J. Integr. Agr. 2022, 21, 3434.

[21] M. Figueroa , K. E. Hammond‐Kosack , P. S. Solomon , Mol. Plant Pathol. 2018, 19, 1523. 29045052 10.1111/mpp.12618 PMC6638159

[22] a) Y. Chen , H. C. Kistler , Z. Ma , Annu. Rev. Phytopathol. 2019, 57, 15; 30893009 10.1146/annurev-phyto-082718-100318

[23] a) R. D. Taylor , A. Saparno , B. Blackwell , V. Anoop , S. Gleddie , N. A. Tinker , L. J. Harris , Proteomics 2008, 8, 2256; 18452225 10.1002/pmic.200700610

[24] D. C. Stenger , G. N. Revington , M. C. Stevenson , D. M. Bisaro , Proc. Natl. Acad. Sci. U.S.A. 1991, 88, 8029. 1896448 10.1073/pnas.88.18.8029 PMC52439

[25] P. Gong , H. Tan , S. Zhao , H. Li , H. Liu , Y. Ma , X. Zhang , J. Rong , X. Fu , R. Lozano‐Duran , F. Li , X. Zhou , Nat. Commun. 2021, 12, 4278. 34257307 10.1038/s41467-021-24617-4 PMC8277811

[26] R. Vanitharani , P. Chellappan , C. M. Fauquet , Trends Plant Sci. 2005, 10, 144. 15749473 10.1016/j.tplants.2005.01.005

[27] M. Aregger , B. K. Borah , J. Seguin , R. Rajeswaran , E. G. Gubaeva , A. S. Zvereva , D. Windels , F. Vazquez , T. Blevins , L. Farinelli , M. M. Pooggin , PLoS Pathog. 2012, 8, e1002941. 23028332 10.1371/journal.ppat.1002941 PMC3460622

[28] S. Wang , S. Ruan , M. Zhang , J. Nie , C. Nzabanita , L. Guo , J. Fungi 2022, 8, 1237. 10.3390/jof8121237 PMC9781238 36547570

[29] Y. Chen , Q. Gao , M. Huang , Y. Liu , Z. Liu , X. Liu , Z. Ma , Sci. Rep. 2015, 5, 12500. 26212591 10.1038/srep12500 PMC4515635

[30] N. Suzuki , L. M. Geletka , D. L. Nuss , J. Virol. 2000, 74, 7568. 10906211 10.1128/jvi.74.16.7568-7577.2000 PMC112278

[31] a) X. Zhang , D. L. Nuss , Proc. Natl. Acad. Sci. U.S.A. 2008, 105, 16749; 18922782 10.1073/pnas.0807225105 PMC2567904

[32] H. Wang , S. Sun , W. Ge , L. Zhao , B. Hou , K. Wang , Z. Lyu , L. Chen , S. Xu , J. Guo , M. Li , P. Su , X. Li , G. Wang , C. Bo , X. Fang , W. Zhuang , X. Cheng , J. Wu , L. Dong , W. Chen , W. Li , G. Xiao , J. Zhao , Y. Hao , Y. Xu , Y. Gao , W. Liu , Y. Liu , H. Yin , et al., Science 2020, 368, eaba5435.32273397

[33] D. L. Nuss , Nat. Rev. Microbiol. 2005, 3, 632. 16064055 10.1038/nrmicro1206

[34] C. A. Cuomo , U. Güldener , J. Xu , F. Trail , B. G. Turgeon , A. D. Pietro , J. D. Walton , L. Ma , S. E. Baker , M. Rep , G. Adam , J. Antoniw , T. Baldwin , S. Calvo , Y. Chang , D. DeCaprio , L. R. Gale , S. Gnerre , R. S. Goswami , K. Hammond‐Kosack , L. J. Harris , K. Hilburn , J. C. Kennell , S. Kroken , J. K. Magnuson , G. Mannhaupt , E. Mauceli , H. W. Mewes , R. Mitterbauer , G. Muehlbauer , et al., Science 2007, 317, 1400. 17823352 10.1126/science.1143708

[35] R. H. Proctor , S. P. McCormick , H. S. Kim , R. E. Cardoza , A. M. Stanley , L. Lindo , A. Kelly , D. W. Brown , T. Lee , M. M. Vaughan , N. J. Alexander , M. Busman , S. Gutierrez , PLoS Pathog. 2018, 14, e1006946. 29649280 10.1371/journal.ppat.1006946 PMC5897003

