# Supplementary material for: A Mycovirus VIGS Vector Confers Hypovirulence to a Plant Pathogenic Fungus to Control Wheat FHB
Source: Adv Sci (Weinh). 2023 Aug 16;10(29):2302606. doi: 10.1002/advs.202302606 (PMC10582431; doi:10.1002/advs.202302606)
Supplement: Supplementary file 1 — Supporting Information [file ADVS-10-2302606-s001.pdf]

ADVANCED SCIENCE
Open Access

Supporting Information

for *Adv. Sci.*, DOI 10.1002/advs.202302606

A Mycovirus VIGS Vector Confers Hypovirulence to a Plant Pathogenic Fungus to Control Wheat FHB

*Lihang Zhang, Shuangchao Wang, Shaojian Ruan, Clement Nzabanita, Yanfei Wang and Lihua Guo**

Supporting Information

Title

A mycovirus VIGS vector confers hypovirulence to a plant pathogenic fungus to control wheat FHB

Lihang Zhang†, Shuangchao Wang†, Shaojian Ruan, Clement Nzabanita, Yanfei Wang, and Lihua Guo*

State Key Laboratory for Biology of Plant Diseases and Insect Pests, Institute of Plant Protection, Chinese Academy of Agricultural Sciences, Beijing, China

† L.Z. and S.W. contributed equally to this work.

***Corresponding author: Lihua Guo**

E-mail: guolihua@caas.cn

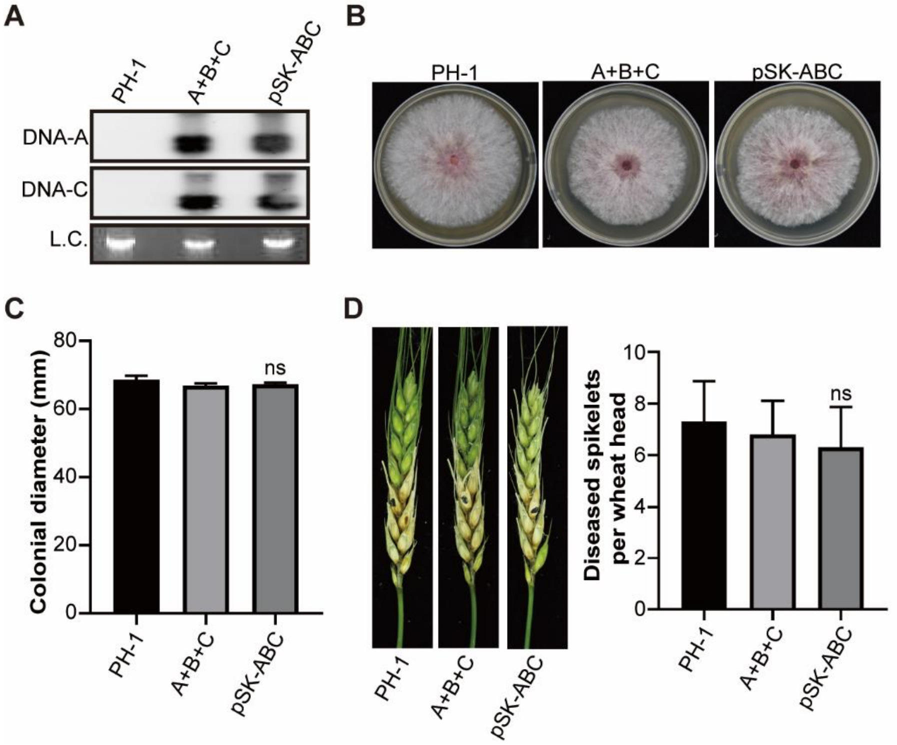

Figure S1. Comparison of colony morphology, colonial diameter, viral DNA accumulation, and virulence between fungal cultures transfected with plasmid vectors A+B+C and pSK-ABC. (A) Southern blot showing viral DNA accumulation of strains PH-1 (VF, as negative control), A+B+C (as positive control), and pSK-ABC. Fungal genomic DNA serves as the loading control (L.C.). The blots show the components of DNA-A and DNA-C, respectively. This experiment was repeated three times with similar results; representative images are shown. **(B)** Colony morphology of strains PH-1, A+B+C, and pSK-ABC after 4 days of culture on PDA in the dark (n = 3). **(C)** Comparison of the colonial diameter among strains PH-1, A+B+C, and pSK-ABC (n = 3). **(D)** FHB symptoms and the number of diseased spikelets per invaded wheat head caused by putting small equisized mycelial plugs into the glume of spikelets (n = 15). Twelve days after inoculation, there were no significant differences in the number of diseased spikelets per invaded wheat head infected with strains A+B+C and pSK-ABC. Error bars represent standard deviation. ns indicates no significance.

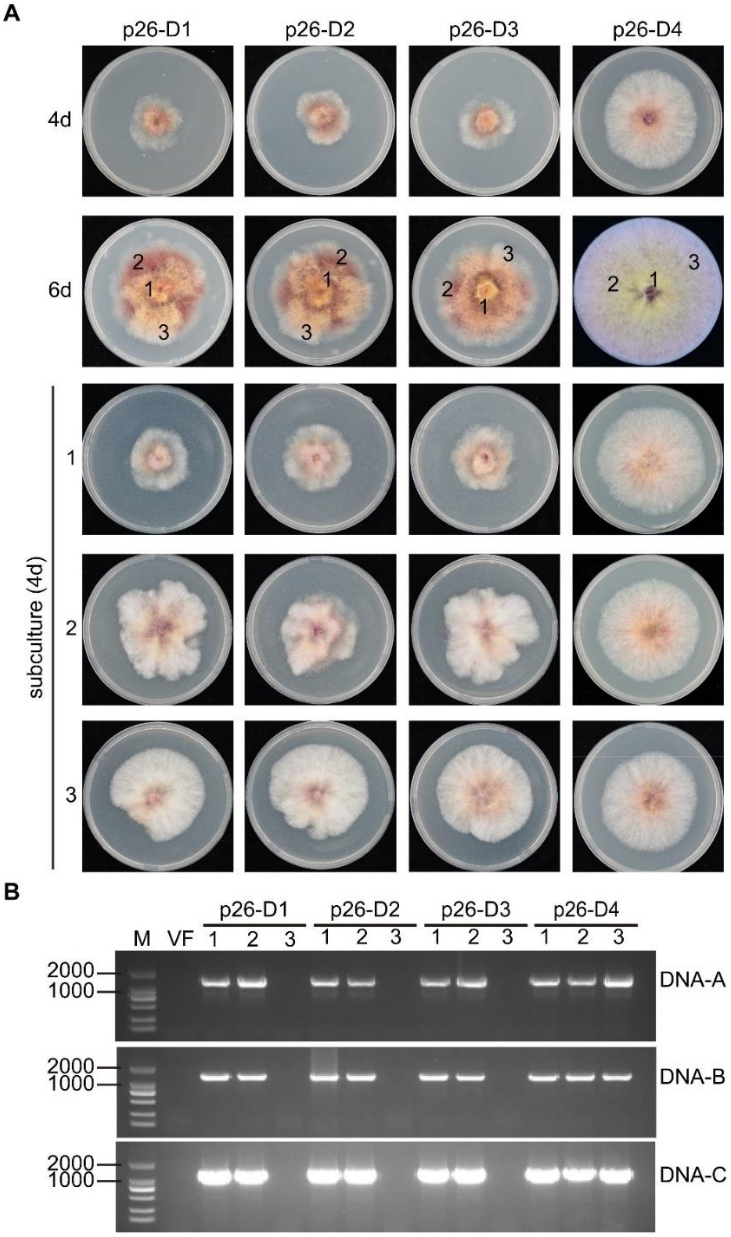

Figure S2. Colony morphology and virus detection of mutants p26-D1, p26-D2, p26-D3, and p26-D4 during culture on PDA. (A) Colony morphology of mutants p26-D1, p26-D2, p26-D3, and p26-D4 was observed after 4 or 6 days of culture and subculture on PDA under dark conditions. **(B)** PCR detection of the viral components DNA-A, DNA-B, and DNA-C in the samples from (A). VF: negative control. This experiment was repeated thrice with similar results. Representative images are shown.

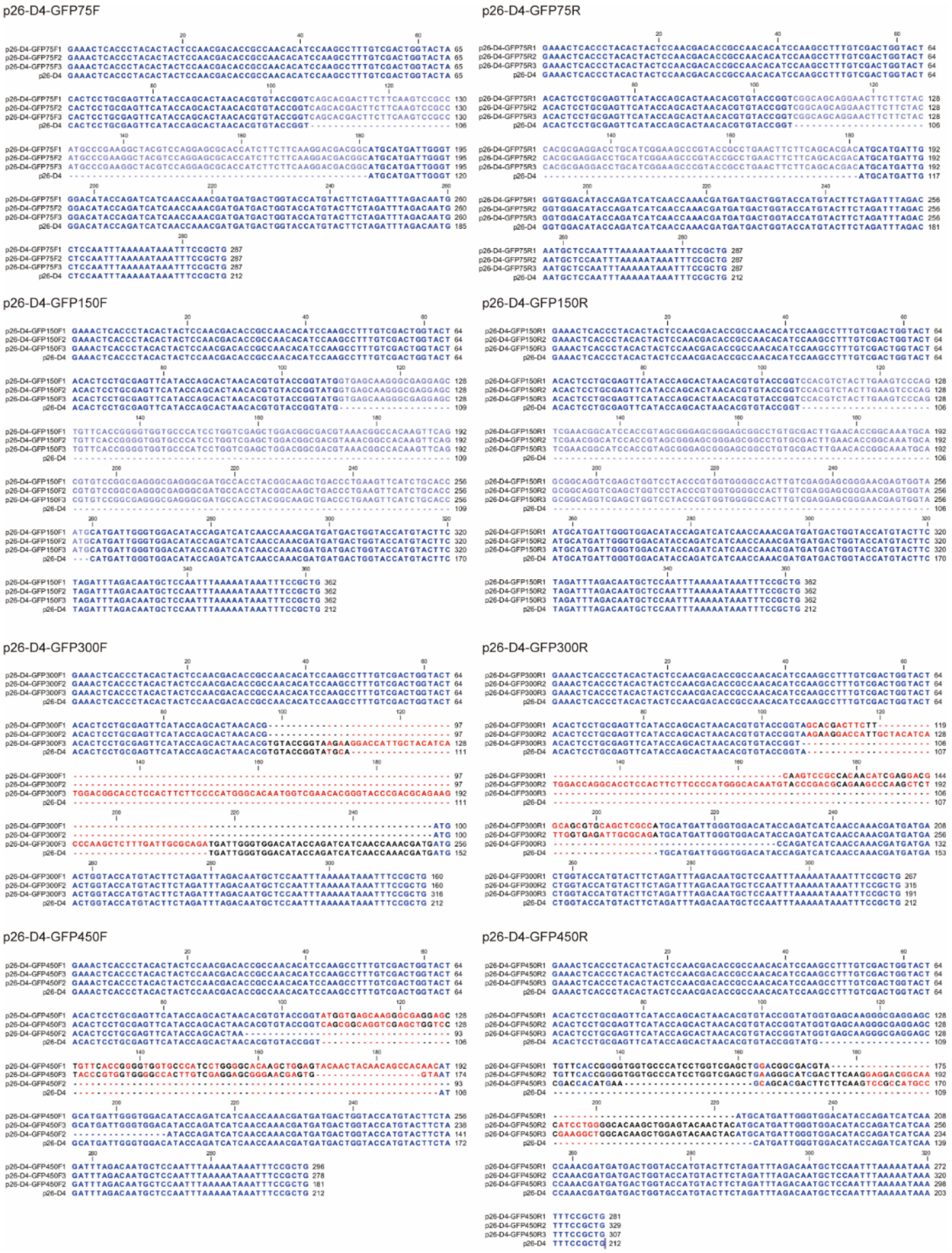

Figure S3. Sequence analysis of the inserted GFP gene fragments and the conjugated viral genome in transfectants infected by p26-D4-GFP75F/R, p26-D4-GFP150F/R, p26-D4-GFP300F/R, and p26-D4-GFP450F/R. There were no changes in the inserted fragments and viral genome sequences in transfectants infected by p26-D4-GFP75F/R and p26-D4-GFP150F/R. The GFP fragments inserted into DNA-C genomes of strains infected by p26-D4-GFP300F/R and p26-D4-GFP450F/R were partially or entirely deleted. This experiment was repeated thrice with similar results. Representative sequences are shown.

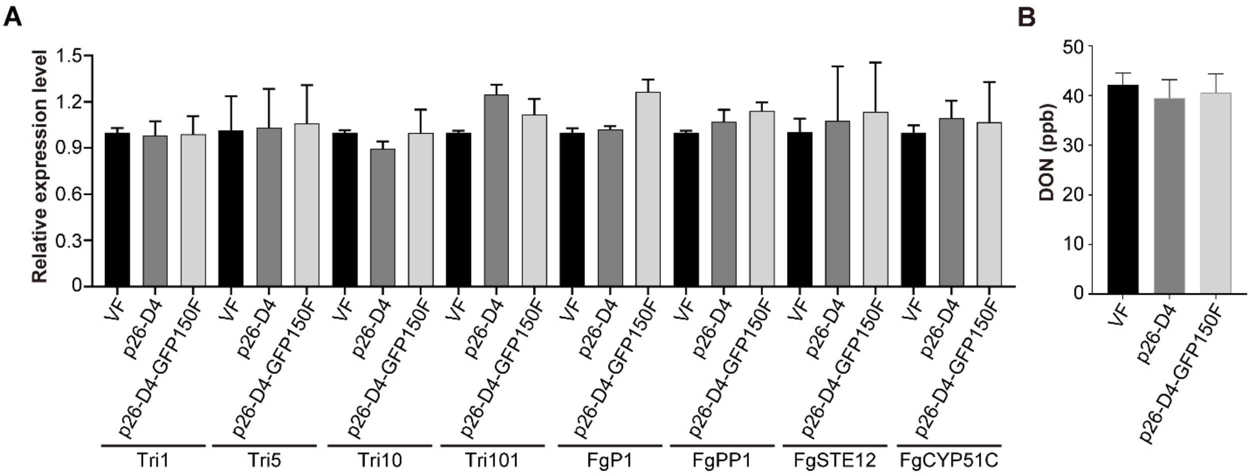

Figure S4. The relative expression levels of eight target genes and DON production analysis in p26-D4-GFP150F-infected strain. (A) The relative expression levels of *Tri1*, *Tri5*, *Tri10*, *Tri101*, *FgP1*, *FgPP1*, *FgSTE12*, and *FgCYP51C* in p26-D4-GFP150F-infected strain. qRT-PCR was carried out with the *EF-1α* transcript levels as an internal control (n = 3). **(B)** DON production analysis in p26-D4-GFP150F-infected strain. The DON production of each strain was determined after growth in mycotoxin induction medium (TBI) for three days (n = 3).

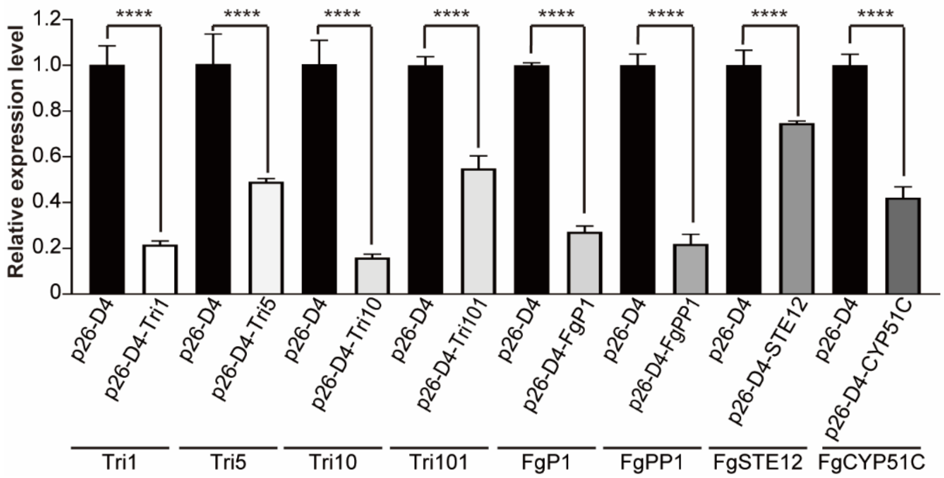

Figure S5. The relative expression level of target genes in the sixth subculture. The expression of *Tri1*, *Tri5*, *Tri10*, *Tri101*, *FgP1*, *FgPP1*, *FgSTE12*, and *FgCYP51C* are downregulated in the strains infected with p26-D4-Tri1, p26-D4-Tri5, p26-D4-Tri10, p26-D4-Tri101, p26-D4-FgP1, p26-D4-FgPP1, p26-D4-FgSTE12 or p26-D4-FgCYP51C, respectively. These samples were sub-cultured six times. qRT-PCR was carried out with the *EF-1α* transcript levels as an internal control (n = 3). Error bars represent standard deviation. An asterisk indicates a statistically significant difference according to the one-way ANOVA (Dunnett's *post-hoc* test). **** $p < 0.0001$.

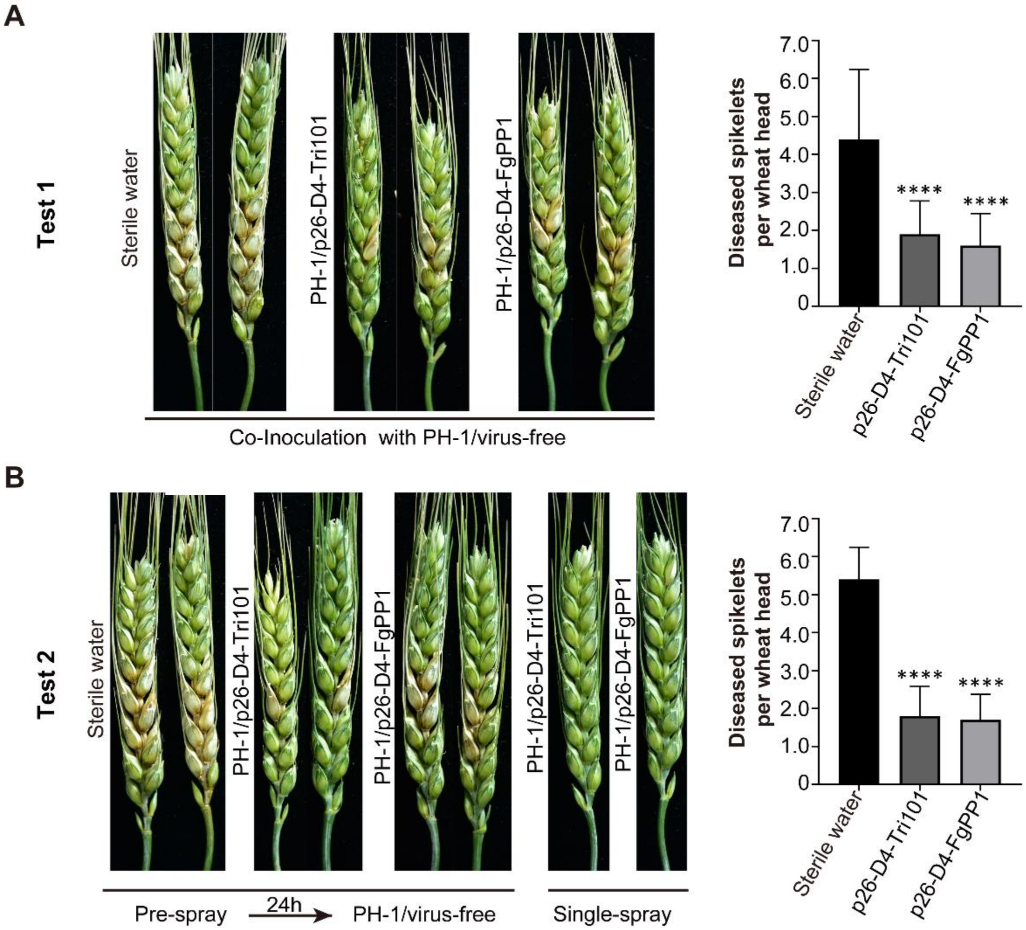

Figure S6. The control of FHB in wheat with hypovirulent strains containing the VIGS vector in the field. FHB symptom and the number of diseased spikelets per invaded wheat head in Test 1 **(A)** and Test 2 **(B)** (n = 15). Test 1: Small equisized mycelial plugs of the two hypovirulent strains were inoculated together with 10 μL of conidial suspension of strain PH-1/*hph* into the glume of a spikelet. Test 2: Hyphal fragment suspensions were sprayed on wheat spikes, followed by inoculation of 10 μL PH-1/*hph* conidial suspension at 24 h post spraying. Infected wheat heads were photographed 12 days after inoculation. Sterile water was used as the negative control. The data was analyzed using GraphPad Prism version 8.0. Error bars represent standard deviation. An asterisk indicates a statistically significant difference according to the one-way ANOVA (Dunnett's *post-hoc* test). **** $p < 0.0001$.

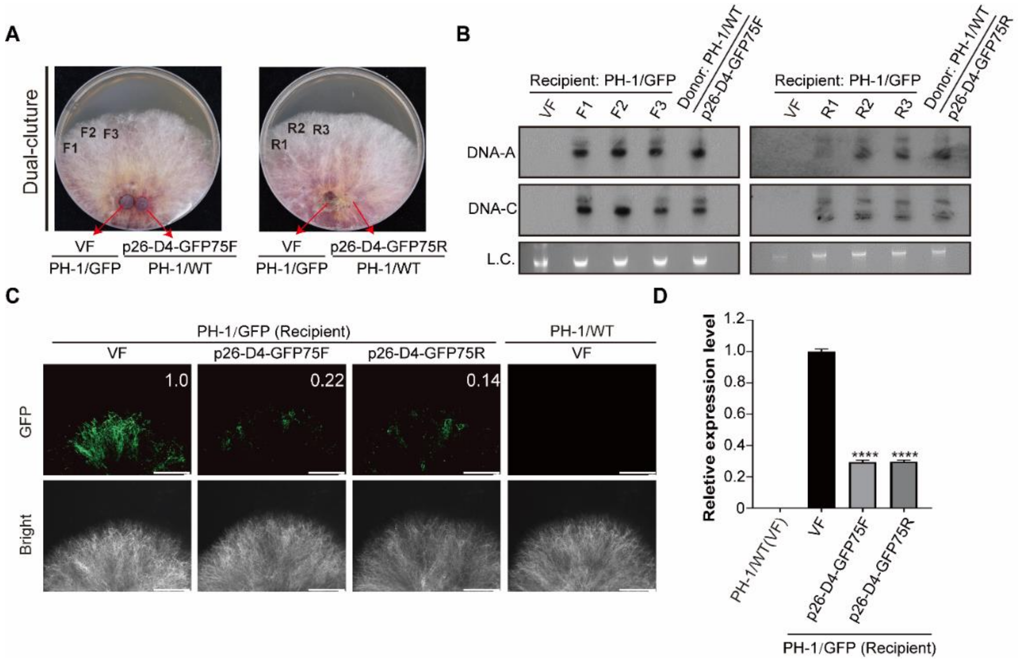

Figure S7. p26-D4-based vectors could silence target genes by viral horizontal transmission via hyphal anastomosis. (A) Viral horizontal transmission via hyphal anastomosis. Strains infected by p26-D4-GFP75F and p26-D4-GFP75R were used as donors, and strain PH-1/GFP as a recipient. **(B)** Southern blot analysis of DNAs extracted from mycelia of strains PH-1/GFP (VF, as negative control), p26-D4-GFP75F/R-infected strains (as positive control) and subcultures (F1-F3, R1-R3) from the recipient sides of dual-cultures between PH-1/GFP and strains infected by p26-D4-GFP75F or p26-D4-GFP75R. Fungal genomic DNA serves as the loading control (L.C.). The blots show the components of DNA-A and DNA-C, respectively. This experiment was repeated three times with similar results; representative images are shown. **(C)** p26-D4-based vector mediated the suppression of the GFP reporter expression by viral horizontal transmission via hyphal anastomosis. The recipients infected by p26-D4-GFP75F and p26-D4-GFP75R are significantly weak in green fluorescence intensity. Values in the respective panels of (C) show the relative intensity of GFP green fluorescence quantified by ImageJ, with virus-free strain PH-1/GFP expressed as 1.0. Scale bars, 5 mm. This experiment was repeated thrice with similar results. Representative images are shown. **(D)** The relative expression level of GFP in recipient strains infected by p26-D4-GFP75F and p26-D4-GFP75R. qRT-PCR was carried out with the *EF-1α* transcript levels as an internal control (n = 3). Error bars represent standard deviation. An asterisk indicates a statistically significant difference according to the one- way ANOVA (Dunnett's *post-hoc* test). **** $p < 0.0001$. ns indicates no significance.

Table S1. All *F. graminearum* strains used in this study.

Strains	Brief description	Reference
PH-1	Wild-type	[34]
PH-1/GFP	PH-1 integrated the enhanced green fluorescent protein (EGFP) gene and the Geneticin-resistance gene (G418)	This study
PH-1/pSK-ABC	Transfectant of the infectious clone pSK-ABC	This study
PH-1/p26-D1	Transfectant of the viral deletion mutant p26-D1, strain PH-1 as a recipient	This study
PH-1/p26-D2	Transfectant of the viral deletion mutant p26-D2, strain PH-1 as a recipient	This study
PH-1/p26-D3	Transfectant of the viral deletion mutant p26-D3, strain PH-1 as a recipient	This study
PH-1/p26-D4	Transfectant of the viral deletion mutant p26-D4, strain PH-1 as a recipient	This study
PH-1/p26-D5	Transfectant of the viral deletion mutant p26-D5, strain PH-1 as a recipient	This study
PH-1(GFP)/p26-D4	Transfectant of the viral deletion mutant p26-D4, strain PH-1/GFP as a recipient	This study
PH-1(GFP)/p26-D4-GFP75F	Transfectant of the VIGS vector p26-D4-GFP75F, strain PH-1/GFP as a recipient	This study
PH-1(GFP)/p26-D4-GFP75R	Transfectant of the VIGS vector p26-D4-GFP75R, strain PH-1/GFP as a recipient	This study
PH-1(GFP)/p26-D4-GFP150F	Transfectant of the VIGS vector p26-D4-GFP150F, strain PH-1/GFP as a recipient	This study
PH-1(GFP)/p26-D4-GFP150R	Transfectant of the VIGS vector p26-D4-GFP150R, strain PH-1/GFP as a recipient	This study
PH-1(GFP)/p26-D4-GFP300F	Transfectant of the VIGS vector p26-D4-GFP300F, strain PH-1/GFP as a recipient	This study

PH-1(GFP)/p26-D4-GFP300R	Transfectant of the VIGS vector p26-D4-GFP300R, strain PH-1/GFP as a recipient	This study
PH-1(GFP)/p26-D4-GFP450F	Transfectant of the VIGS vector p26-D4-GFP450F, strain PH-1/GFP as a recipient	This study
PH-1(GFP)/p26-D4-GFP450R	Transfectant of the VIGS vector p26-D4-GFP450R, strain PH-1/GFP as a recipient	This study
PH-1/p26-D4-GFP75F	Transfectant of the VIGS vector p26-D4-GFP75F, strain PH-1 as a recipient	This study
PH-1/p26-D4-GFP75R	Transfectant of the VIGS vector p26-D4-GFP75R, strain PH-1 as a recipient	This study
PH-1/p26-D4-Tri1	Transfectant of the VIGS vector p26-D4-Tri1, strain PH-1 as a recipient	This study
PH-1/p26-D4-Tri5	Transfectant of the VIGS vector p26-D4-Tri5, strain PH-1 as a recipient	This study
PH-1/p26-D4-Tri10	Transfectant of the VIGS vector p26-D4-Tri10, strain PH-1 as a recipient	This study
PH-1/p26-D4-Tri101	Transfectant of the VIGS vector p26-D4-Tri101, strain PH-1 as a recipient	This study
PH-1/p26-D4-FgP1	Transfectant of the VIGS vector p26-D4-FgP1, strain PH-1 as a recipient	This study
PH-1/p26-D4-FgPP1	Transfectant of the VIGS vector p26-D4-FgPP1, strain PH-1 as a recipient	This study
PH-1/p26-D4-FgSTE12	Transfectant of the VIGS vector p26-D4-FgSTE12, strain PH-1 as a recipient	This study
PH-1/p26-D4-FgCYP51C	Transfectant of the VIGS vector p26-D4-FgCYP51C, strain PH-1 as a recipient	This study

Table S2. Genes involved in trichothecene biosynthesis or pathogenicity in *F. graminearum*.

Gene name	Brief description	Silencing efficiency	Reference
Tri1 (FGSG_00071)	*Tri1* encodes cytochrome P450 oxygenases and catalyzes hydroxylation of C-7 and C-8 of isotrichodermin and calonectrin during the biosynthesis of type B trichothecenes. The gene disruptants no longer produced 15-acetyldeoxynivalenol, but accumulated calonectrin and 3-deacetylcalonectrin.	83%	[22]
Tri5 (FGSG_03537)	*Tri5* encodes a trichodiene synthase, which cyclizes farnesyl pyrophosphate into trichodiene, the first step in trichothecene biosynthesis. *Tri5* deletion mutant could not produce trichothecene and exhibited reduced virulence on some hosts.	84%	[22b, 23b]
Tri10 (FGSG_03538)	*Tri10* is a regulatory gene in trichothecene mycotoxin-producing *Fusarium* species and required for trichothecene biosynthesis. The expression levels of most trichothecene genes such as *Tri1*, *Tri3*, *Tri4*, *Tri5*, *Tri8*, *Tri11*, *Tri101* are regulated by *Tri10*. DON production and pathogenicity were significantly reduced in *Tri10* deletion mutants.	85%	[22a, 23c]
Tri101 (FGSG_07896)	*Tri101* encodes a C-3 acetyltransferase that is responsible for the conversion of isotrichodermol to isotrichodermin in the biosynthesis pathway of *Fusarium* trichothecene. *Tri101* is an integral enzyme in the progression of the biosynthetic steps.	82%	[22b, 23a]

Biosynthetic pathways for the trichothecene deoxynivalenol in *F. graminearum*: [35]

Farnesyl diphosphate $\xrightarrow{\textit{Tri5}}$ Trichodiene $\xrightarrow{\textit{Tri4}}$ Isotrichotriol $\xrightarrow{\text{nonenzymatic}}$ Isotrichodermol $\xrightarrow{\textit{Tri101}}$ Isotrichodermin $\xrightarrow{\textit{Tri11}}$ 15-decalonectrin $\xrightarrow{\textit{Tri3}}$ Calonectrin $\xrightarrow{\textit{Tri1}}$ 3,15-di-O-acetyldeoxynivalenol $\xrightarrow{\textit{Tri8}}$ 15-acetyldeoxynivalenol $\xrightarrow{?}$ Deoxynivalenol

FgP1 (FGSG_12164)	*FgP1* is a WOR1-like gene. Deletion of the *FgP1* in *F. graminearum* results in greatly reduced pathogenicity and loss of trichothecene toxin accumulation in infected wheat plants and in vitro. *FgP1* is also involved in the developmental processes of conidium formation and sexual reproduction.	69%	[23d]
FgPP1 (FGSG_07233)	*FgPP1* encodes an essential phosphatase that plays an important role in hyphal growth, development, plant infection and secondary metabolism synthesis.	63%	[23e, 23i]
FgSTE12 (FGSG_07310)	*FgSTE12* is a orthologue of *Saccharomyces cerevisiae STE12*, which is a key transcription factor activated by Fus3/Kss1. The *FgSTE12* deletion mutant was impaired in virulence and in the secretion of cellulase and protease.	82%	[23f]
FgCYP51C (FGSG_11024)	*FgCYP51C* is one of the three *CYP51* paralogues that is required for full virulence to host wheat ears. In addition, wheat spikelets inoculated with *FgCYP51C* deletion mutant contained five-fold less DON at 10 dpi than those inoculated with PH-1.	32%	[23j,h]

Table S3. The gene fragment sequences used to obtain hypovirulent strains in this work.

Gene name	Gene fragment sequence
Tri1 FGSG_00071	AAACACAACTGGTATCTCTTCCAAGGCTTGACAGACCATTTCG CTGTCATGGCTCTCATCACCAGTTTGCAGGATGTCCGGCTCGAC ATGCTGGCATATTTTGTTGCCTTTCTCGTCGTAGTATCCGTCGTA
Tri5 FGSG_03537	TTATTGAATAACTGTTACCAGTACAACCTTGCCATCATGGAAAA CTTTCCCACCGAGTATTTTCTCAACACTAGCGTGCGCCTTCTCG AGTATATTCGATACCGAGACAGCAATTACACCCGAGAGGAGCG
Tri10 FGSG_03538	ATGGATTTCCCAAAGCCTAGACAAGTCCGAGAGACGAGCCTGT TGATGTACTACCTAGACGTCGTGTTTCCTCTGCAATGCATCAAC CCAAACAACAATTGTCTGGGAAAGAGAGAGTGGCTGTTGACTA
Tri101 FGSG_07896	GATTTGTACTCTGTTCCCAAGCGTCATCTTTCTCAGCGCAGCAC TTCTATAATTTAGCGGCCTCACCTTCTGTAACACCAACACCAAG TGATTTACAAACACCACCAAAATGGCTTTCAAGATACAGCTCG
Fgp1 FGSG_12164	ATGGCAAATTCAGTGCTCACAGCAACCTATGAGGGTTATATCA GGGACACCGCCGATGCTCTCAGAATCTTCGAGGCTTGCTTGAC TGGCAGCCTTACACACACCGCTCGACGACCACACGATCGGGAA
FgPP1 FGSG_07233	ACCATCTGCTTGCTCCTCGCCTACAAGATCAAGTACCCCGAAA ACTTCTTCATCCTTCGAGGTAACCACGAGTGTGCCTCCATCAAC CGTATTTATGGATTCTACGACGAGTGCAAGCGTCGCTATAACAT
FgSTE12 FGSG_07310	ACATTCGCCGATTCCTGCTGCCCACAGGTGAATATGTCTCATGC ATTCTCTGGAACAACCTCTTCCACATTTCTGGCACCGACATCGT GCGATGCTTGTCCTTCCGATTCCAGGCTTTCGGCCGACCTGTCA
FgCYP51C FGSG_11024	ATGGAATCGCTCTACGAGACTCTGCGGACTCTACCGCTCTCAGT CTCAATCCCTCTAACAACCAGCATCATCATCATCTTGTCCATCG TCACCAACGTGGTCAAACAATTATGGTTTCCCAACCCACATCGT

Table S4. All primers used in this study.

Primer name	Primer sequence	Reference
p26-D1-F	AGGATGCATTACAACTCGCTTACAGCGGAGTTATGCCTT	Used for p26-D1 construction
p26-D1-R	AGGACCGGTCATCTTTATATTGTAAAAAATATTTGTAAC	
p26-D2-F	AGGATGCATAACAACTTTTCTCCGAGCCAAATCAAGGGG	Used for p26-D2 construction
p26-D2-R	AGGACCGGTGGCTTCGTTGGAGGGGAATGTTTCCAGTAT	
p26-D3-F	AGGATGCATCATCTCCACAACAATCCCGACCCACATGAC	Used for p26-D3 construction
p26-D3-R	AGGACCGGTGCCAGAAGGGGTCAGATGTGGCTGAGCAAA	
p26-D4-F	AGGATGCATGATTGGGTGGACATACCAGATCATCAACCA	Used for p26-D4 construction
p26-D4-R	AGGACCGGTACACGTGTTAGTGCTGGTATGAACTCGCAG	
p26-D5-F	AGGATGCATTAAAAATAAATTTCCGCTGCCTAAATCTGC	Used for p26-D5 construction
p26-D5-R	AGGACCGGTAGGATCATTTAAATTATGTACAATTGAATT	
p26-D4-GFP75F-F	CCCACCGGTCAGCACGACTTCTTC	Used for sense 75 bp GFP fragment amplification
p26-D4-GFP75F-R	CCAATGCATGCCGTCGTCCTTGAAG	
p26-D4-GFP75R-F	CCCACCGGTGCCGTCGTCCTTGAAG	Used for antisense 75 bp GFP fragment amplification
p26-D4-GFP75R-R	CCAATGCATCAGCACGACTTCTTC	
p26-D4-GFP150F-F	CCCACCGGTATGGTGAGCAAGGGCGAG	Used for sense 150 bp GFP fragment amplification
p26-D4-GFP150F-R	CCAATGCATGGTGCAGATGAACTTC	
p26-D4-GFP150R-F	CCCACCGGTGGTGCAGATGAACTTC	Used for antisense 150 bp GFP fragment amplification
p26-D4-GFP150R-R	CCAATGCATATGGTGAGCAAGGGCGAG	
p26-D4-GFP300F-F	CCCACCGGTCAGCACGACTTCTTCAAG	

p26-D4-GFP300F-R	CCAATGCATGGCGAGCTGCACGCTG	Used for sense 300 bp GFP fragment amplification
p26-D4-GFP300R-F	CCCACCGGTGGCGAGCTGCACGCTGC	Used for antisense 300 bp GFP fragment amplification
p26-D4-GFP300R-R	CCAATGCATCAGCACGACTTCTTCAAG	
p26-D4-GFP450F-F	CCCACCGGTATGGTGAGCAAGGGCGAG	Used for sense 450 bp GFP fragment amplification
p26-D4-GFP450F-R	CCAGTTGTGGCTGTTGTAGTTGTAC	
p26-D4-GFP450R-F	CCCACCGGTGTTGTGGCTGTTGTAG	Used for antisense 450 bp GFP fragment amplification
p26-D4-GFP450R-R	CCAATGCATATGGTGAGCAAGGGCGAG	
p26-D4-Tri1-F	CAGCACTAACACGTGTACCGGTAAACACAACTGGTATCTC	Used for sense 150 bp *Tri1* fragment amplification
p26-D4-Tri1-R	GTATGTCCACCCAATCATGCATCGGGGCCAGCTTCTTTCGTAC	
p26-D4-Tri5-F	CAGCACTAACACGTGTACCGGTTTATTGAATAACTGTTAC	Used for sense 150 bp *Tri5* fragment amplification
p26-D4-Tri5-R	GTATGTCCACCCAATCATGCATATAGTGCAAATTCTCGATGC	
p26-D4-Tri10-F	CAGCACTAACACGTGTACCGGTATGGATTTCCCAAAGCCTAG	Used for sense 150 bp *Tri10* fragment amplification
p26-D4-Tri10-R	GTATGTCCACCCAATCATGCATAGGCCGCGCAGAGGTCAG	
p26-D4-Tri101-F	CAGCACTAACACGTGTACCGGTGATTTGTACTCTGTTCCC	Used for sense 150 bp *Tri101* fragment amplification
p26-D4-Tri101-R	GTATGTCCACCCAATCATGCATGGTAGCTGGCCGAGGGTGTC	
p26-D4-FgP1-F	CAGCACTAACACGTGTACCGGTATGGCAAATTCAGTGCTCAC	Used for sense 150 bp *FgP1* fragment amplification
p26-D4-FgP1-R	GTATGTCCACCCAATCATGCATGCTTGTAATCAGGGTTGAGC	
p26-D4-FgPP1-F	CAGCACTAACACGTGTACCGGTACCATCTGCTTGCTCCTCGCC	Used for sense 150 bp *FgPP1* fragment amplification
p26-D4-FgPP1-R	GTATGTCCACCCAATCATGCATGAAAGTCTTCCACAACTTG	
p26-D4-FgCYP51C-F	CAGCACTAACACGTGTACCGGTATGGAATCGCTCTACGAGACTC	

p26-D4-FgCYP51C-R	GTATGTCCACCCAATCATGCATATGGAATACAACGGGTGGACG	Used for sense 150 bp *FgCYP51C* fragment amplification
p26-D4-FgSTE12-F	CAGCACTAACACGTGTACCGGTACATTCGCCGATTCCTGCTGC	Used for sense 150 bp *FgSTE12* fragment amplification
p26-D4-FgSTE12-R	GTATGTCCACCCAATCATGCATCGAATTTCTTCGAGTTCTTGAC	
qPCR-EF1α-F	GAAGTTCGAGAAGGAAGC	Used for qPCR amplification
qPCR-EF1α-R	ATGACGGTGACATAGTAG	
qPCR-GFP-F	CGTAAACGGCCACAAGTTCA	Used for qPCR amplification
qPCR-GFP-R	CTTCATGTGGTCGGGGTAGC	
qPCR-Tri1-F	GCTCGTGCAGTCTCAGAAGT	Used for qPCR amplification
qPCR-Tri1-R	ACCTCCTTGATCAGTGCTGC	
qPCR-Tri5-F	TGAGGGATGTTGGATTGAGCAGTAC	Used for qPCR amplification
qPCR-Tri5-R	TGCTTCCGCTCATCAAACAGGT	
qPCR-Tri10-F	CTACAAAGGCTACCGACAGACGA	Used for qPCR amplification
qPCR-Tri10-R	ATCCGTCAAGTCTTCCCATCTCATT	
qPCR-Tri101-F	GCGTGCGTCTCGAAAGAATC	Used for qPCR amplification
qPCR-Tri101-R	GGTAGATGGGTCCGCATCAG	
qPCR-FgCYP51C-F	CGTCCACCCGTTGTATTCCA	Used for qPCR amplification
qPCR-FgCYP51C-R	AACGAGCATTGGAGCAGTCA	
qPCR-Fgp1-F	CTACGATCACCGACCACAGG	Used for qPCR amplification
qPCR-Fgp1-R	GACATGAGCTCCATCCGAGG	
qPCR-FgPP1-F	CGAAATGGCAGACCAACACG	Used for qPCR amplification

qPCR-FgPP1-R	CCGAGGAAGAGGTAGTTGGC	
qPCR-STE12-F	ATGCTTCTTTGGAGGAGCCC	Used for qPCR amplification
qPCR-STE12-R	GCTCGTAGAGCGACTGAGAC	
Probe A-F	TGGGAAGTAGGCGTGATT	Used for Southern blot detection of DNA-A genome
Probe A-R	CACACCAACCATCCTTGA	
Probe B-F	GGCAATCCGCAAACACAT	Used for Southern blot detection of DNA-B genome
Probe B-R	CTCCGTCTTCAACAACGCA	
Probe C-F	GTATGTCCACCCAATCAGG	Used for Southern blot detection of DNA-C genome
Probe C-R	CCCTTCTGGCAACAACTT	
DNA-A-1F	GACCAATCAACTTCCGCA	Used for PCR detection of DNA-A genome
DNA-A-1R	GATGATACGCAACCATTC	
DNA-B-1F	TTATCACCGTATCCGTCGG	Used for PCR detection of DNA-B genome
DNA-B-1R	AACTCCTCCGTCTTCAAC	
DNA-C-1F	CGGAGTTATGCCTTCTATTCTG	Used to clone the complete sequence of DNA-C and for PCR detection of DNA-C genome in mutants p26-D3 and p26-D4
DNA-C-1R	CTGTAAGCGAGTTGTAGGC	
DNA-C-2F	TCATCTCCACAACAATCCC	Used for PCR detection of DNA-C genome in mutants p26-D1 and p26-D2
DNA-C-2R	CACGTGTTAGTGCTGGTATG	
